# Lactate‐Activated GPR132‐Src Signal Induces Macrophage Senescence and Aggravates Atherosclerosis Under Diabetes

**DOI:** 10.1002/advs.202500141

**Published:** 2025-06-10

**Authors:** Xiaofeng Ge, Shuying Wang, Zhaokai Li, Jing Yu, Binbin Liu, Ruiying Wang, Shichen Bu, Nawsher wan, Yan Wang, Cuilian Dai, Yijun Lin

**Affiliations:** ^1^ Xiamen Cardiovascular Hospital of Xiamen University, School of Medicine Fujian Branch of National Clinical Research Center for Cardiovascular Diseases Xiamen University Xiamen 361016 China; ^2^ CAS Key Laboratory of Nutrition Metabolism and Food Safety Shanghai Institute of Nutrition and Health University of Chinese Academy of Sciences Chinese Academy of Sciences Shanghai 200031 China

**Keywords:** atherosclerosis, diabetes, lactate, macrophage, senescence

## Abstract

Diabetes is widely acknowledged as a significant risk factor for atherosclerosis, facilitating plaque formation through various mechanisms. Although both conditions are linked to the aging process, the relationship among cellular senescence, diabetes, and atherosclerosis remains inadequately understood. This study presents evidence that elevated glucose levels expedite the progression of atherosclerosis by promoting macrophage senescence. Increased glucose levels are shown to induce senescence in macrophages, which enhances the uptake of oxidized low‐density lipoprotein (ox‐LDL) and facilitates the formation of foam cells. This mechanism is driven by lactate production via glycolysis, which activates the lactate receptor GPR132, thereby promoting macrophage senescence. The activation of GPR132 is implicated in mediating senescence and lipid uptake through Src phosphorylation. The deletion of GPR132 markedly reduces macrophage senescence and atherosclerosis in mouse models. Furthermore, saracatinib, a specific Src inhibitor, has been demonstrated to effectively alleviate diabetic atherosclerosis in experimental settings. In clinical samples, elevated plasma lactate levels and the activation of the GPR132‐Src pathway in peripheral blood mononuclear cells (PBMCs) are positively associated with coronary stenosis. These findings propose a potential mechanism through which diabetes accelerates atherosclerosis via the lactate‐GPR132‐Src pathway, underscoring macrophage senescence as a pivotal target in the context of diabetic atherosclerosis.

## Graphical abstract

Elevated lactate in diabetes activates the GPR132‐Src pathway in macrophages, inducing macrophage senescence and further enhancing foam cell formation. This mechanism exacerbates atherosclerotic progression.

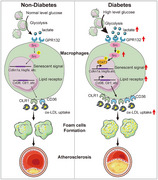

## 1 Introduction

Atherosclerotic cardiovascular disease (ASCVD) has emerged as a predominant cause of mortality globally, posing a significant threat to millions of individuals annually.^[^ ^1^ ^]^ Atherosclerotic lesions are characterized by a lifelong accumulation of lipids, inflammatory cells and necrotic cell debris underneath endothelial cells (ECs), initiated by the infiltration of oxidized lipoproteins into the subendothelial space of arteries.^[^ ^2^ ^]^ The activation of ECs and vascular smooth muscle cells promotes the recruitment of circulating monocytes, which, upon lipid uptake, differentiate into lipid‐laden foamy macrophages. These macrophages are considered central to the initiation and progression of atherosclerotic plaques, with M1 macrophages predominating in early lesions through the secretion of pro‐inflammatory cytokines, while M2 macrophages are associated with plaque stabilization and regression.^[^ ^3^ ^]^ The presence of these macrophages exacerbates lesion formation and sustains the inflammatory microenvironment within the plaque.^[^ ^4^ ^]^

Diabetes mellitus is an important risk factor for ASCVD.^[^ ^5^ ^]^ Individuals with diabetes are 2 to 4 times more likely to experience atherosclerosis and its associated complications.^[^ ^5^, ^6^ ^]^ Several hypotheses suggest that advanced glycation end‐products (AGEs) play a critical role in this process by inducing pro‐inflammatory responses or modifying extracellular matrix components,^[^ ^7^ ^]^ and exosomes in diabetic conditions^[^ ^8^ ^]^ or mitochondrial stress^[^ ^9^ ^]^ play important roles.

Cellular senescence, defined as a permanent cessation of the cell cycle, is associated with the activation of the p16^INK4a^/Rb and p19^ARF^/p53 pathways, as well as metabolic dysregulation and the emergence of pro‐inflammatory phenotypes.^[^ ^10^ ^]^ Advanced atherosclerotic plaques contain cells exhibiting senescence markers, which may serve as a significant source of inflammatory cytokines.^[^ ^4^ ^]^ Senescent foam cells are detrimental throughout all stages of atherosclerosis, although their specific roles remain poorly understood. An early investigation indicated that senescence markers are predominantly co‐localized with CD68, suggesting that macrophages within the plaque exhibit a senescent phenotype.^[^ ^11^ ^]^ The prevailing evidence indicates that the senescence‐associated secretory phenotype (SASP) is integral to the mechanism by which cellular senescence contributes to atherosclerosis.^[^ ^2^, ^4^ ^]^ Furthermore, it is noteworthy that senescence and diabetes are closely interconnected, as the diabetic microenvironment appears to augment the burden of senescent cells, potentially triggered by glucose and AGEs.^[^ ^12^ ^]^

Lactate serves as a vital metabolic regulator, functioning both as a significant energy source for the tricarboxylic acid (TCA) cycle and as a signaling molecule that influences metabolic disorders.^[^ ^13^ ^]^ Elevated lactate levels have been documented in individuals with diabetes,^[^ ^14^ ^]^ potentially contributing to increased insulin resistance.^[^ ^15^ ^]^ Additionally, high blood lactate levels were detected in patients with established carotid atherosclerosis.^[^ ^16^ ^]^ In the present study, we demonstrate that diabetes significantly influences macrophage senescence and foam cell formation via lactate. Elevated glucose levels stimulate lactate‐mediated paracrine signaling that activates GPR132‐Src pathways; the deletion of GPR132 or inhibition of Src was found to alleviate atherosclerosis. These findings suggest that the lactate‐GPR132‐Src axis may play a pivotal role in promoting macrophage senescence and the uptake of ox‐LDL, thereby accelerating the progression of atherosclerosis in the context of diabetes.

## 2 Results

### 2.1 Diabetes‐Aggravated Atherosclerotic Progress in Patients and Mice and Promoted Metabolic Dysregulation and Senescence in Peripheral Macrophages

**Figure 1:**
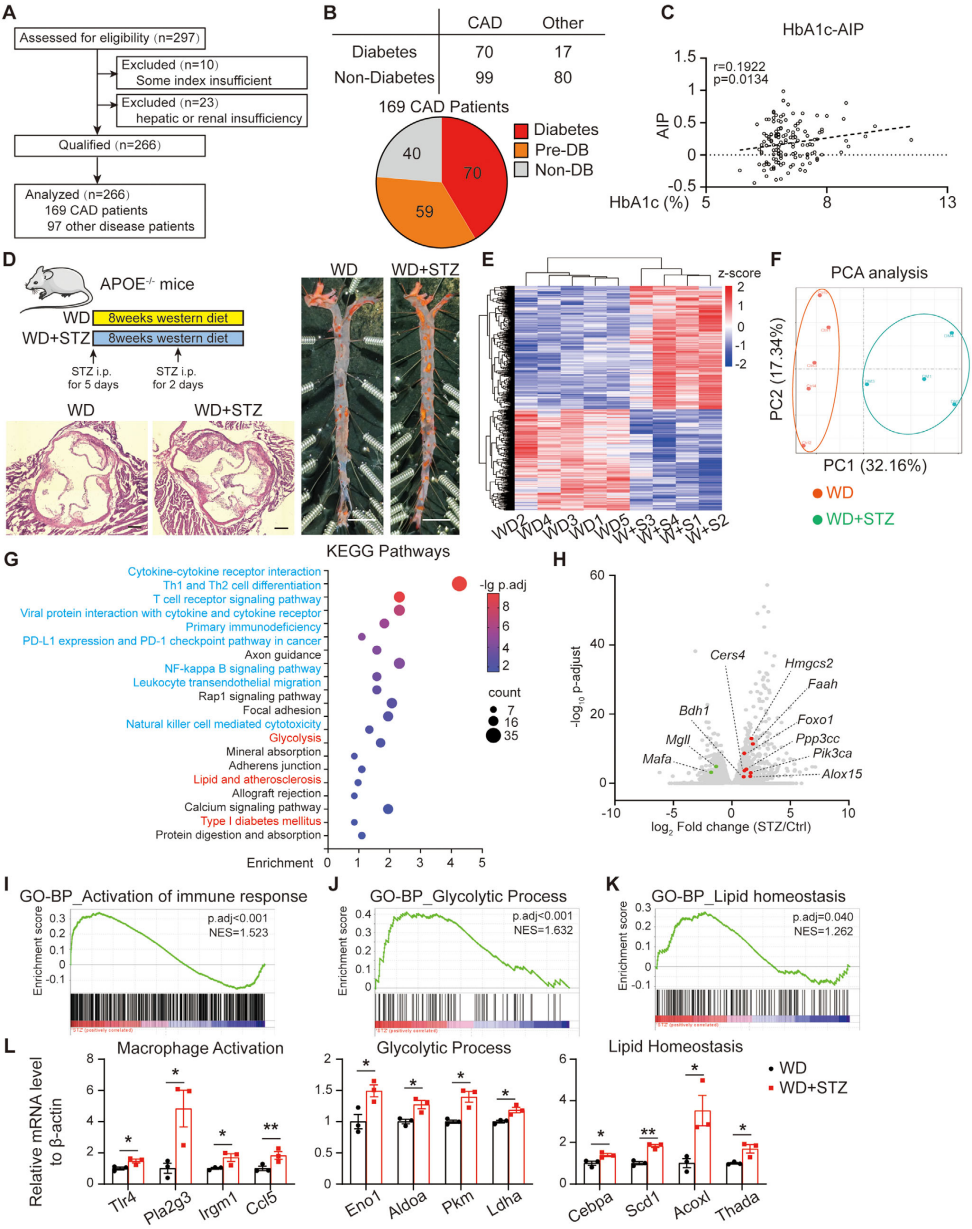
Diabetes aggravates atherosclerosis and reprograms the metabolism of peripheral macrophages. A) A diagram for the clinical data analysis flow. B) Groups of 266 patients according to diabetes and coronary arteriosclerosis. C) Correlation analysis of HbA1c and Atherogenic index of plasma (AIP). The correlations were analyzed with linear regression. D) A diagram for the mouse model of diabetic atherosclerosis. 4 mg kg^−1^ STZ or citric acid was injected in the first and fifth weeks of the experiment. The mice were totally fed with the Western diet for 8 weeks. Representative images of Oil Red O stained aorta were shown in the right panel and the arterial roots with H&E staining were shown below. (E) The heatmap shows the differently expressed genes of mouse peritoneal macrophages. Samples are ordered by hierarchical clustering similarity. F) The PCA analysis of the RNA‐sequencing data of mouse peritoneal macrophages. G) Bubble charts to show the KEGG analysis of the differently expressed genes. H) Volcano plots to show the differently expressed genes. The marked plots are significantly changed genes belonging to the lipid and glucose homeostasis pathway, according to GO analysis. I–K) GSEA analysis of three pathways: the activation of immune response (I), glycolytic process (J), and lipid homeostasis pathway (K). (L) Relative mRNA levels of genes of macrophage activation, glycolytic process, and lipid homeostasis pathways. *n* = 3 for each group. The data were analyzed with a student t‐test. *p < 0.05, **p < 0.01, ***p < 0.001.

We noted that 76% of patients were either diabetic or pre‐diabetic among the 169 hospitalized patients with coronary arteriosclerotic cardiomyopathy within the cardiology division‐5 of Xiamen Cardiovascular Hospital in January 2024 (**Figure** **1**B). Atherogenic index of plasma (AIP), a recognized marker for assessing atherosclerosis risk,^[^ ^17^ ^]^ demonstrated a positive correlation with HbA1c levels (Figure 1C), while low‐density lipoprotein cholesterol (LDL‐C) and apolipoprotein B (APOB) also increased in tandem with HbA1c levels (Figure S1B, Supporting Information). Further investigations in mice were undertaken by a streptozotocin (STZ)‐induced diabetic model established in Apoe^−/−^ mice, as referenced in previous studies.^[^ ^5^, ^18^ ^]^ Diabetic condition exacerbated the accumulation of blood lipids (Figure S2C,E, Supporting Information) and worsened atherosclerosis compared to the control group, as evidenced by increased plaque volume and macrophage infiltration (Figure 1D; Figure S2F,G, Supporting Information).

The majority of immune cells accumulating in intimal lesions originate from blood,^[^ ^19^ ^]^ especially the macrophages accumulated to become foam cells. To elucidate their role in the diabetic milieu and its effect on arterial plaque formation, especially the role of myeloid cells, peritoneal macrophages were isolated for RNA sequencing. The analysis revealed a distinct separation between macrophages from diabetic and nondiabetic mice, identifying 978 upregulated and 955 downregulated genes (Figure 1E,F), indicative of significant transcriptional reprogramming induced by diabetes. The most affected pathways were related to inflammation (blue) and metabolic reprogramming (red), as determined by KEGG analysis (Figure 1G). While the association between a hyperglycemic environment and inflammation or glucose metabolism is well established, alterations in lipid metabolism were also observed, with several metabolic genes exhibiting significant changes (Figure 1H). These alterations in metabolic pathways were further validated by Gene Set Enrichment Analysis (GSEA) (Figure 1I–K), and some genes of the pathways were confirmed by qPCR (Figure 1L). The results emphasize that diabetes induces metabolic reprogramming of peritoneal macrophages.

**Figure 2:**
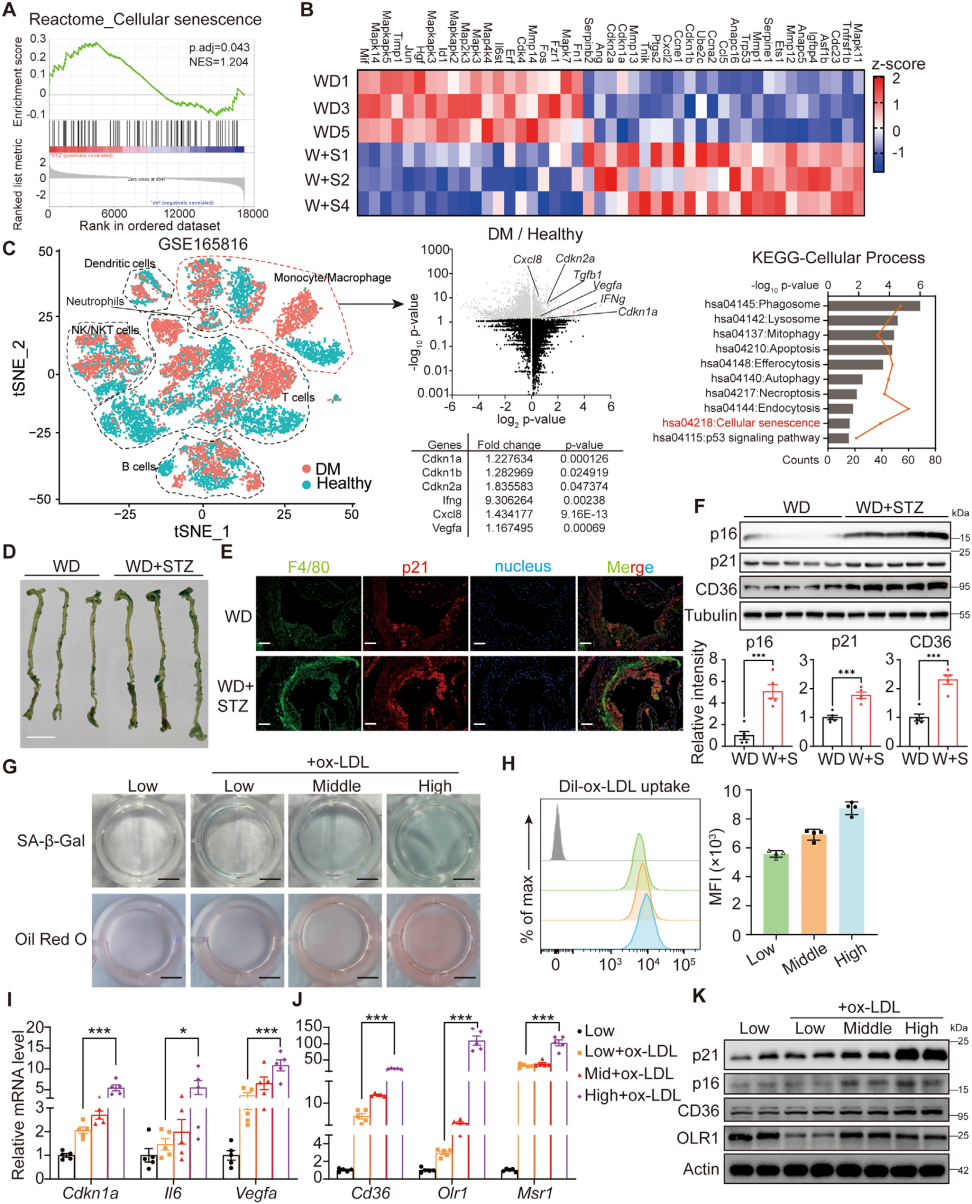
High glucose level promotes macrophage senescence in vivo and in vitro. (A) GSEA analysis of the cellular senescence pathway. B) Heatmap to show the genes belonging to the cellular senescence pathway, according to KEGG. C) Analysis of single‐cell RNA‐sequencing data of GSE165816. The volcano plots of differently expressed genes in monocytes/ macrophages, the expression data of some SASP genes, and the KEGG analysis of the genes were shown in the right panel. D) Representative images of β‐Gal stained aorta. E) Representative images of the arterial roots with F4/80 (green) and p21 (Red) staining. F) Western blotting to detect p16, p21, and CD36 in the peritoneal macrophages of the mice as in Figure 1. Quantification of p16, p21, and CD36 relative to tubulin is shown in the below panel. G) Representative whole‐well images of Oil Red O and β‐Gal stained RAW264.7 cells. Low: treated with DMEM with 1.5 mg mL^−1^ glucose for 48 h. Low+ox‐LDL: pre‐treated with DMEM with 1.5 mg mL^−1^ glucose for 24 h followed by 40 mg mL^−1^ ox‐LDL addition for 24 h; Middle/Mid+ox‐LDL: pre‐treated with DMEM with 3 mg mL^−1^ glucose for 24 h followed by 40 mg mL^−1^ ox‐LDL addition for 24 hours; High+ox‐LDL: pre‐treated with DMEM with 4.5 mg mL^−1^ glucose for 24 h followed by 40 mg mL^−1^ ox‐LDL addition for 24 h. H) Flow cytometry analysis of Dil‐ox‐LDL uptake by the RAW264.7 cells under treatment. The pre‐treatment was the same as in G but the Dil‐ox‐LDL was treated for 4 hours with 10 mg mL^−1^. I, J) Relative mRNA levels of lipid uptake genes (I) and SASP genes (H) in the RAW264.7 cells as in G. *n* = 5 for each group. K) Western blotting to detect p21, p16, OLR1, and CD36 in the RAW264.7 cells as in G. The quantitative data of E and F were analyzed with student t‐test, and the quantitative data of I and J were analyzed with one‐way ANOVA. *p < 0.05; **p < 0.01; ***p < 0.001.

Importantly, gene analysis revealed a positive correlation between the cellular senescence pathway and diabetes (**Figure** **2**A; Figure S2H, Supporting Information). Notable aging markers including *Cdkn1a*, *Cdkn2a*, *Igfbp4*, and *Ccl5* were significantly upregulated in cells from diabetic mice (Figure 2B). Analysis of a single‐cell dataset (GSE165816), which contains RNA profiles of peripheral immune cells from both healthy and diabetic patients, revealed that monocytes/macrophages, rather than other cell types, in diabetic patients exhibited elevated expression levels of SASP genes (Figure 2C), suggesting the presence of a senescent phenotype in peripheral macrophages. Western blotting further confirmed that peritoneal macrophage senescence was exacerbated by diabetes (Figure 2F). Given the accumulation of peripheral cells in plaques, we conducted aorta staining for β‐galactosidase and p21 (Figure 2D,E; Figure S2I, Supporting Information), which further demonstrated that diabetes promotes monocytes/macrophages senescence in vivo, particularly in the atherosclerotic plaque.

### 2.2 Elevated Glucose Promotes ox‐LDL Uptake by Macrophage by inducing Cellular Senescence

Senescence is thought to be an important regulator of the atherosclerosis process,^[^ ^4^ ^]^ particularly associated with lipid metabolism disorder.^[^ ^20^ ^]^ Previous studies have indicated that senescent cells exhibit an increased capacity for lipid uptake,^[^ ^21^ ^]^ and our research also demonstrated an upregulation of CD36, a principal lipid receptor in macrophages, in immune cells derived from diabetic Apoe^−/−^ mice (Figure 2F). To replicate this regulatory environment in vitro, we pre‐treated RAW264.7 macrophage cells with varying concentrations of glucose prior to exposure to ox‐LDL for 24 h. The results indicated a dose‐dependent increase in both glycolytic process and ox‐LDL uptake correlating with elevated glucose levels (Figure 2G,H; Figure S3A,B, Supporting Information), which may result from the increase of CD36 and OLR1 (Figure 2I,K). Meanwhile, high‐glucose conditions were associated with elevated macrophage senescence (Figure 2G,J,K), suggesting that elevated glucose levels may exacerbate atherosclerosis through mechanisms involving macrophage senescence and enhanced ox‐LDL uptake. Similar results that high‐glucose accelerated senescence and lipid uptake have also been demonstrated in human‐derived monocytes THP‐1 (Figure S3C–G, Supporting Information).

**Figure 3:**
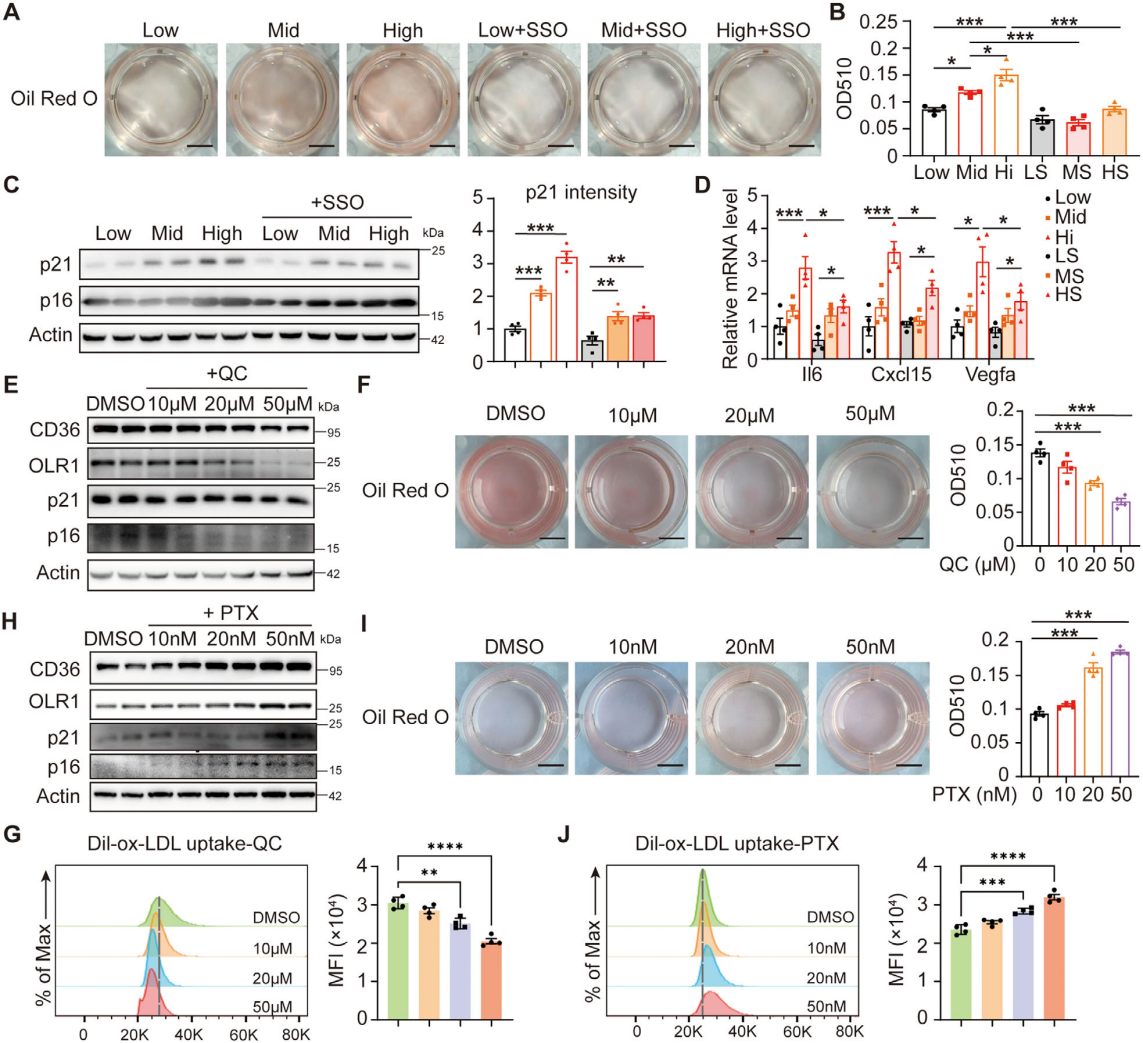
High levels of glucose promoted macrophage ox‐LDL uptake through cell senescence. A) Representative whole‐well images of Oil Red O stained RAW264.7 cells, which were pre‐treated with DMEM with different concentrations of glucose and 50 µmol L^−1^ SSO for 6 h followed by 24 h stimulation with 40 mg mL^−1^ ox‐LDL. Low, DMEM with 1.5 mg mL^−1^ glucose; Mid, DMEM with 3 mg mL^−1^ glucose; High, DMEM with 4.5 mg mL^−1^ glucose. B) Quantification of the Oil Red O staining of the cells in A. *n* = 4 for each group. C) Western blotting to detect p21 and p16 in the RAW264.7 cells as in A. Quantification of p21 relative to β‐actin from two independent experiments was shown in the right panel. D) Relative mRNA levels of SASPs in the RAW264.7 cells as in A. *n* = 4 for each group. E) Western blotting to detect p16, p21, CD36, and OLR1 in the RAW264.7 cells pre‐treated with 4.5 mg mL^−1^ glucose DMEM and different concentrations of quercetin (QC) for 12 h followed by 24 h stimulation with 40 mg mL^−1^ ox‐LDL. F) Representative whole‐well images of Oil Red O stained RAW264.7 cells as in E. Quantification of the Oil Red O staining of the cells in the right panel. *n* = 4 for each group. G) Flow cytometry analysis of Dil‐ox‐LDL uptake by the RAW264.7 cells under 24‐h QC treatment followed by 4‐h 10 mg mL^−1^ Dil‐ox‐LDL treatment. H) Western blotting to detect p16, p21, CD36, and OLR1 in the RAW264.7 cells pre‐treated with 1.5 mg mL^−1^ glucose DMEM and different concentrations of paclitaxel (PTX) for 12 hours followed by 24 h stimulation with 40 mg mL^−1^ ox‐LDL. I) Representative whole‐well images of Oil Red O stained RAW264.7 cells as in H. Quantification of the Oil Red O staining of the cells in the right panel. *n* = 4 for each group. J) Flow cytometry analysis of Dil‐ox‐LDL uptake by the RAW264.7 cells under 24‐h PTX treatment followed by 4‐h 10 mg mL^−1^ Dil‐ox‐LDL treatment. All the quantitative data were analyzed with one‐way ANOVA. *p < 0.05; **p < 0.01; ***p < 0.001, ****p < 0.0001.

To further elucidate the interplay between glucose levels, macrophage senescence, and ox‐LDL uptake, we initially pre‐treated RAW264.7 cells with Sulfo‐N‐succinimidyl oleate (SSO), a CD36 inhibitor by directly binding with CD36 bur not affecting its expression (Figure S3H, Supporting Information). SSO significantly reduced ox‐LDL uptake of macrophages (**Figure** **3**A,B); however, cellular senescence continued to be exacerbated by elevated glucose levels (Figure 3C,D), indicating that glucose‐influenced macrophage senescence regardless of ox‐LDL uptake. Subsequently, we explored whether senescence itself affected ox‐LDL uptake. The flavonoid quercetin, recognized for its anti‐aging properties, was found to decrease the expression of CD36 and OLR1 in a dose‐dependent manner (Figure 3E), leading to a ≈50% reduction in ox‐LDL uptake at a concentration of 50 µM (Figure 3F,G). This suggests that inhibiting macrophage senescence may reduce ox‐LDL uptake. Furthermore, the induction of cellular senescence using paclitaxel significantly increased ox‐LDL uptake in a dose‐dependent manner (Figure 3H–J). The findings collectively indicate that elevated glucose levels enhance macrophage CD36 expression and ox‐LDL uptake through the promotion of senescence.

### 2.3 Lactate is a Mediator of Macrophage Senescence

**Figure 4:**
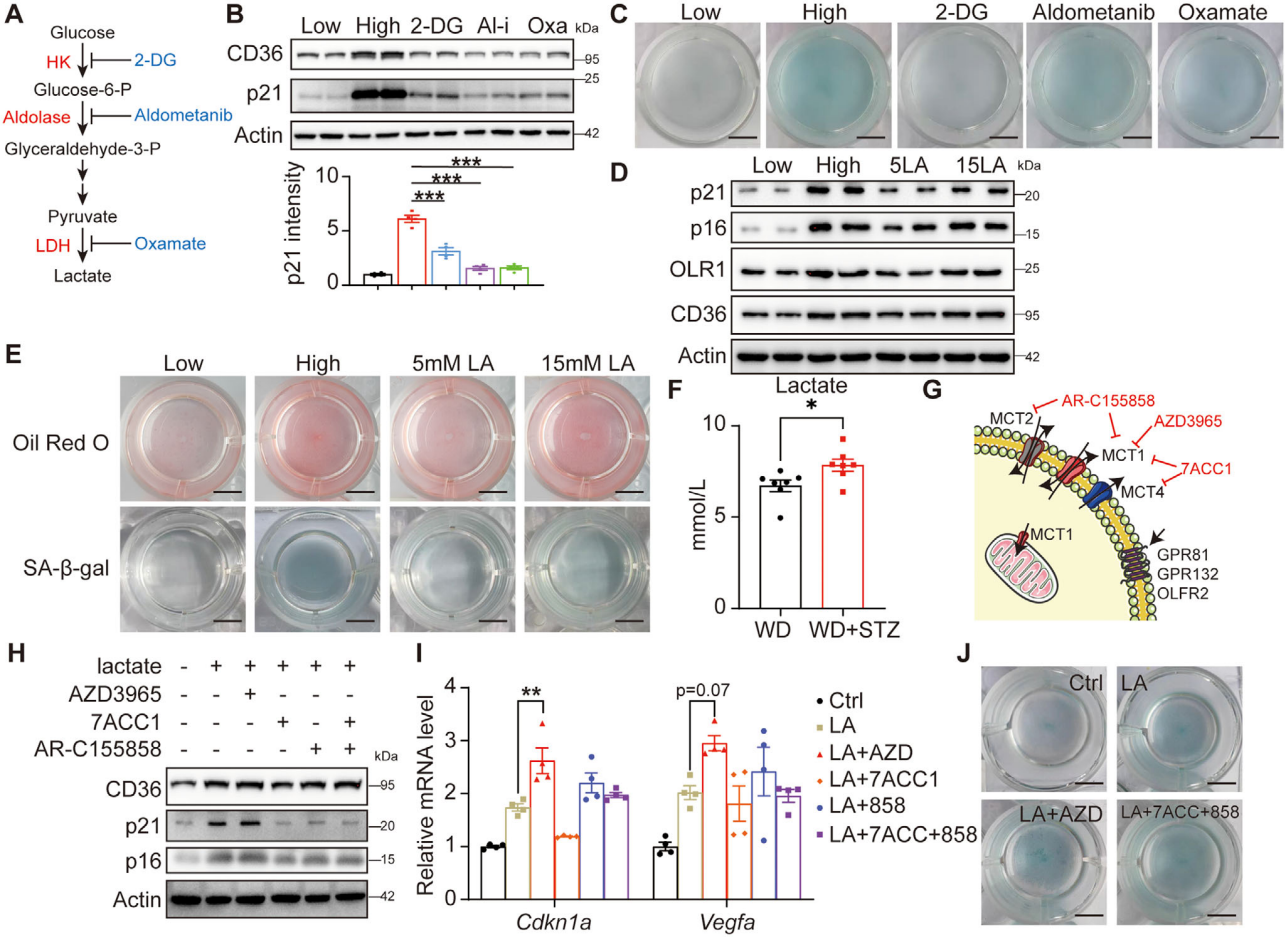
Lactate produced from glycolysis promotes senescence in an extracellular way. A) A diagram to show the key steps of glycolysis and the inhibitors. B) Western blotting to detect p21 and CD36 in the RAW264.7 cells pre‐treated with 1.5 mg mL^−1^ glucose (Low), 4.5 mg mL^−1^ glucose (High), 4.5 mg mL^−1^ glucose with 2 mg mL^−1^ 2‐DG (2‐DG), 4.5 mg mL^−1^ glucose with 1 µmol L^−1^ aldometanib (Al‐i), 4.5 mg mL^−1^ glucose with 10 mmol L^−1^ oxamic acid sodium (Oxa) for 12 h followed by 24 h stimulation with 40 mg mL^−1^ ox‐LDL. Quantification of p21 relative to β‐actin from two independent experiments is shown below, *n* = 4 for each group. (C) Representative whole‐well images of β‐Gal stained RAW264.7 cells as in B. (D) Western blotting to detect p21, p16, CD36 and OLR1 in the RAW264.7 cells pre‐treated with 1.5 mg mL^−1^ glucose (Low), 4.5 mg mL^−1^ glucose (High), 1.5 mg mL^−1^ glucose with 5 mmol L^−1^ lactate (5LA), 1.5 mg mL^−1^ glucose with 15 mmol L^−1^ lactate (15LA) for 12 h, followed by 24 h stimulation with 40 mg mL^−1^ ox‐LDL. (E) Representative whole‐well images of β‐Gal and Oil Red O stained RAW264.7 cells as in D. (F) The levels of lactate in the plasma of the mice in Figure 1. *n* = 7 for each group. (G) A diagram to show lactate transport and recognition in cells. AZD3965 specific inhibits MCT1; 7ACC1 targets both MCT1 and MCT4; AR‐C155858 targets MCT1 and MCT2. (H) Western blotting to detect p21, p16, and CD36 in the RAW264.7 cells cultured with 1.5 mg mL^−1^ glucose, respectively pre‐treated with 10 mmol L^−1^ lactate, 1 µmol L^−1^ AZD3965, 0.5 mmol L^−1^ 7ACC1, 0.1 µmol L^−1^ AR‐C155858 for 12 h, followed by 24 h stimulation with 40 mg/mL ox‐LDL. I) Relative mRNA levels of Cdkn1a and Vegfa in the RAW264.7 cells as in H. J) Representative whole‐well images of β‐Gal stained RAW264.7 cells as in H. The quantitative data of B and I were analyzed with one‐way ANOVA, while the data of F were analyzed with student t‐tests. *p < 0.05; **p < 0.01; ***p < 0.001.

Elevated glucose levels have been shown to enhance glycolysis in macrophages (Figure 1J; Figure S3A,C, Supporting Information). As the Warburg effect is activated in senescent cells,^[^ ^22^ ^]^ metabolic irregularities may contribute to cellular senescence. A series of inhibitors targeting various stages of glycolysis were assessed in the condition of high glucose to identify the specific step responsible for macrophage senescence (**Figure** **4**A). Notably, all inhibitors were found to reduce senescence (Figure 4B,C; Figure S4A, Supporting Information), indicating that either downstream products or the glycolytic pathway itself may be involved in this phenomenon.

As the final by‐product of glycolysis, lactate is released by macrophages exposed to ox‐LDL due to heightened glycolytic activity.^[^ ^23^ ^]^ Exposing RAW264.7 cells to lactate resulted in a dose‐dependent increase in both cellular senescence and lipid uptake (Figure 4D,E; Figure S4B, Supporting Information), indicating a potential role for lactate in these processes. It is noteworthy that diabetes can also elevate blood lactate levels (Figure 4F), implying that lactate may be a contributing factor to macrophage senescence promoted by diabetes.

Lactate can function intracellularly or extracellularly, whose balance is regulated by its transport of MCTs and SMCTs^[^ ^24^ ^]^ (Figure 4G). Even though MCT1 mediates the bi‐directional transport of lactate in some cells, it mainly mediates lactate influx in macrophages while MCT4 mediates lactate efflux (Data not shown). Interestingly, RNA levels of all transporters were significantly increased under high glucose conditions, while only MCT1 exhibited upregulation at the protein level (Figure S4C,D, Supporting Information), suggesting an enhanced influx of lactate. To investigate whether lactate preferentially regulates cellular senescence through extracellular or intracellular signaling, various inhibitors were utilized to treat RAW264.7 cells under the condition of low glucose with 10 mM lactate (Figure 4G). Notably, treatment with AZD3965, a specific inhibitor of MCT1, significantly increased extracellular lactate concentration (Figure S4E, Supporting Information) and further accelerated macrophage senescence (Figure 4H–J), while the bi‐directional channel inhibitors 7ACC1 showed no effect. These findings underscore the critical role of MCT1 in lactate transport in macrophages and suggest that lactate may promote macrophage senescence through extracellular mechanisms.

### 2.4 GPR132 is a Key Lactate Receptor to Facilitate Macrophage Senescence and Atherosclerosis

**Figure 5:**
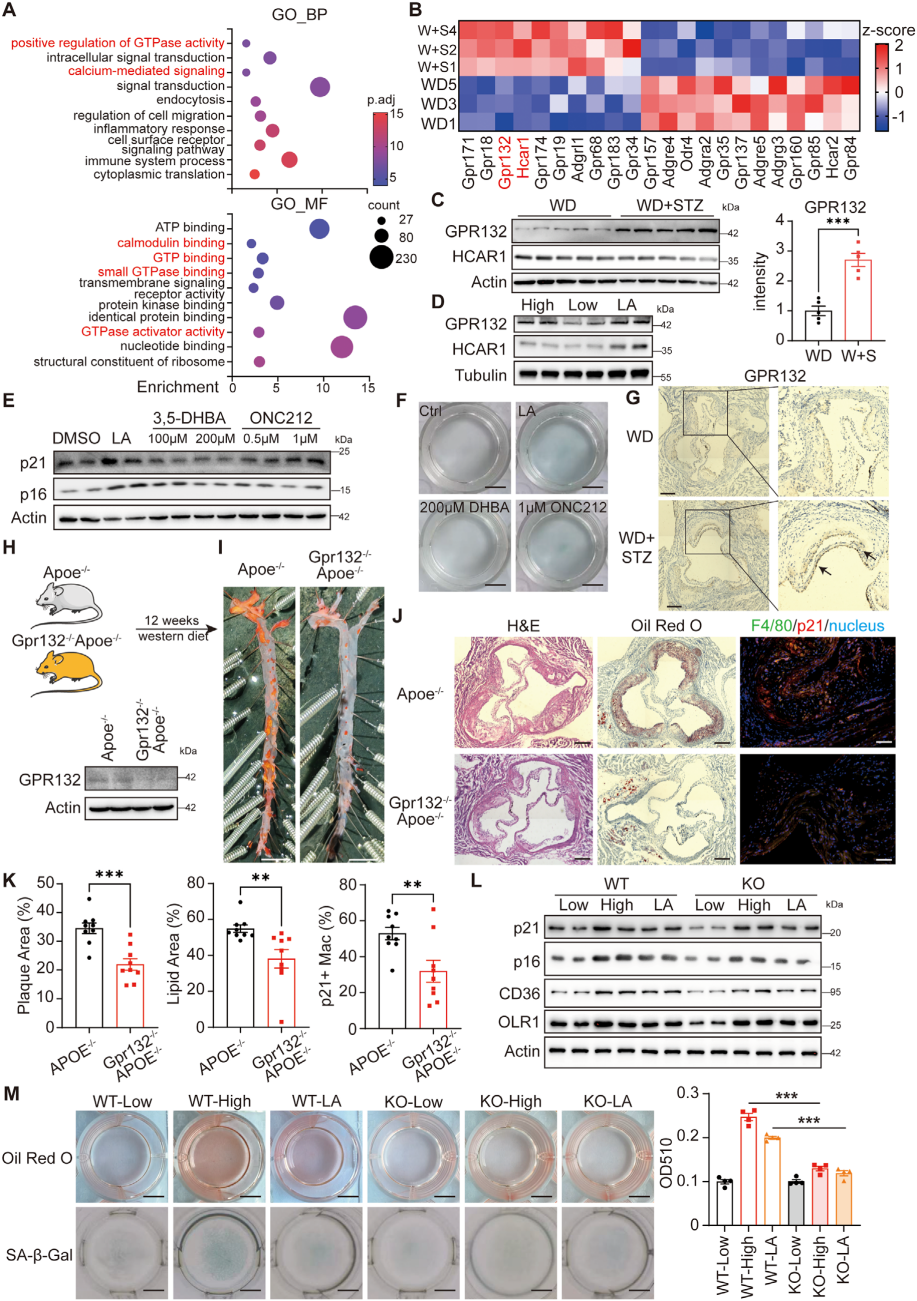
Lactate‐mediated macrophage senescence through GPR132. A) Bubble charts to show the GO analysis of the differently expressed genes, with the RNA‐seq data shown in Figure 2. B) Heatmap to show the expression of GPCR genes. C) Western blotting to detect GPR132 and HCAR1 in mouse peritoneal macrophages as in Figure 2. Quantification of GPR132 was shown in the right panel. D) Western blotting to detect GPR132 and HCAR1 in the RAW264.7 cells pre‐treated with DMEM with 4.5 mg/mL glucose (High), 1.5 mg mL^−1^ glucose (Low), 1.5 mg mL^−1^ glucose with 10 mmol L^−1^ lactate (LA) for 24 h. E) Western blotting to detect p21 and p16 in the RAW264.7 cells cultured with 1.5 mg mL^−1^ glucose DMEM, respectively pre‐treated with DMSO (DMSO), 10 mmol L^−1^ lactate (LA), different concentrations of 3,5‐DHBA or ONC212 for 24 h, followed by 24 h stimulation with 40 mg mL^−1^ ox‐LDL. (F) Representative images of β‐Gal stained RAW264.7 cells in E. G) Representative images of GPR132 immunohistochemistry of the aortic roots in the mice as in Figure 1. H) Western blotting to detect the deletion of GPR132 in BMDMs. I) Representative images of Oil Red O stained aorta. J) Representative images of the arterial roots with H&E staining, Oil Red O staining, F4/80 (green), and p21 (red) staining. K) Quantification of the plaques in I‐J. *n* = 9 for each group. L) Western blotting to detect p21, p16, OLR1 and CD36 in the BMDMs from Apoe^−/−^ (WT) and Gpr132^−/−^Apoe^−/−^ (KO) mice, pre‐treated with 1.5 mg/mL glucose DMEM (low), 4.5 mg mL^−1^ glucose DMEM (high), and 1.5 mg/mL glucose DMEM with10mmol/L lactate (LA) for 24 h, followed by 24 h stimulation with 40 mg/mL ox‐LDL. M) Representative whole‐well images of Oil Red O and β‐gal stained RAW264.7 cells as in L. Quantification of Oil Red O were shown in the right panel. *n* = 4 for each group. The quantitative data in C and K were analyzed with student t‐tests, and the data in M were analyzed with one‐way ANOVA. *p < 0.05; **p < 0.01; ***p < 0.001.

Gene Ontology (GO) analysis of sequencing data indicated significant modifications in GPCR signaling pathways, with GPR132 and HCAR1 being recognized as lactate receptors (**Figure** **5**A,B).^[^ ^25^ ^]^ Western blot analysis demonstrated an increase in GPR132 levels, but not HCAR1, in peripheral cells and RAW264.7 macrophages subjected to elevated glucose or lactate conditions (Figure 5C,D). To further elucidate the receptor responsible for inducing senescence, RAW264.7 cells were pre‐treated with 3, 5‐DHBA, and ONC212, specific agonists for HCAR1 and GPR132, respectively. As only ONC212 enhanced both the expressions of p21 and p16 and β‐Gal staining in a manner comparable to lactate (Figure 5E,F; Figure S5A, Supporting Information), the results indicated that GPR132 was the key receptor for macrophage senescence. Additionally, increased expression of GPR132 was observed in the arterial roots of diabetic Apoe^−/−^ mice (Figure 5G), and GPR132 is predominantly expressed in monocytes within the bloodstream (Figure S5B, Supporting Information).

Then, Gpr132^−/−^Apoe^−/−^ mice were generated to investigate the role of GPR132 in atherosclerosis in vivo (Figure 5H). The absence of GPR132 led to a significant reduction in atherosclerotic plaques (Figure 5I; Figure S5E, Supporting Information), evidenced by a 31% decrease in plaque burden (Figure 5J,K). Importantly, there was a notable decrease in p21^+^ macrophages within the aortic roots (Figure 5J,K) and a reduction in β‐gal staining in the aorta (Figure S5G, Supporting Information), suggesting that GPR132 functions as a promoter of cellular senescence in the context of atherosclerosis. Gpr132^−/−^ BMDMs exhibited increased resistance to senescence induced by elevated glucose or lactate, as well as reduced uptake of ox‐LDL (Figure 5L,M), further substantiating the hypothesis that GPR132 serves as a critical lactate receptor in macrophage senescence.

### 2.5 GPR132 Induced Src Phosphorylation Mediates Macrophage Senescence and Saracatinib is a Potential Medicine for Diabetic Atherosclerosis

**Figure 6:**
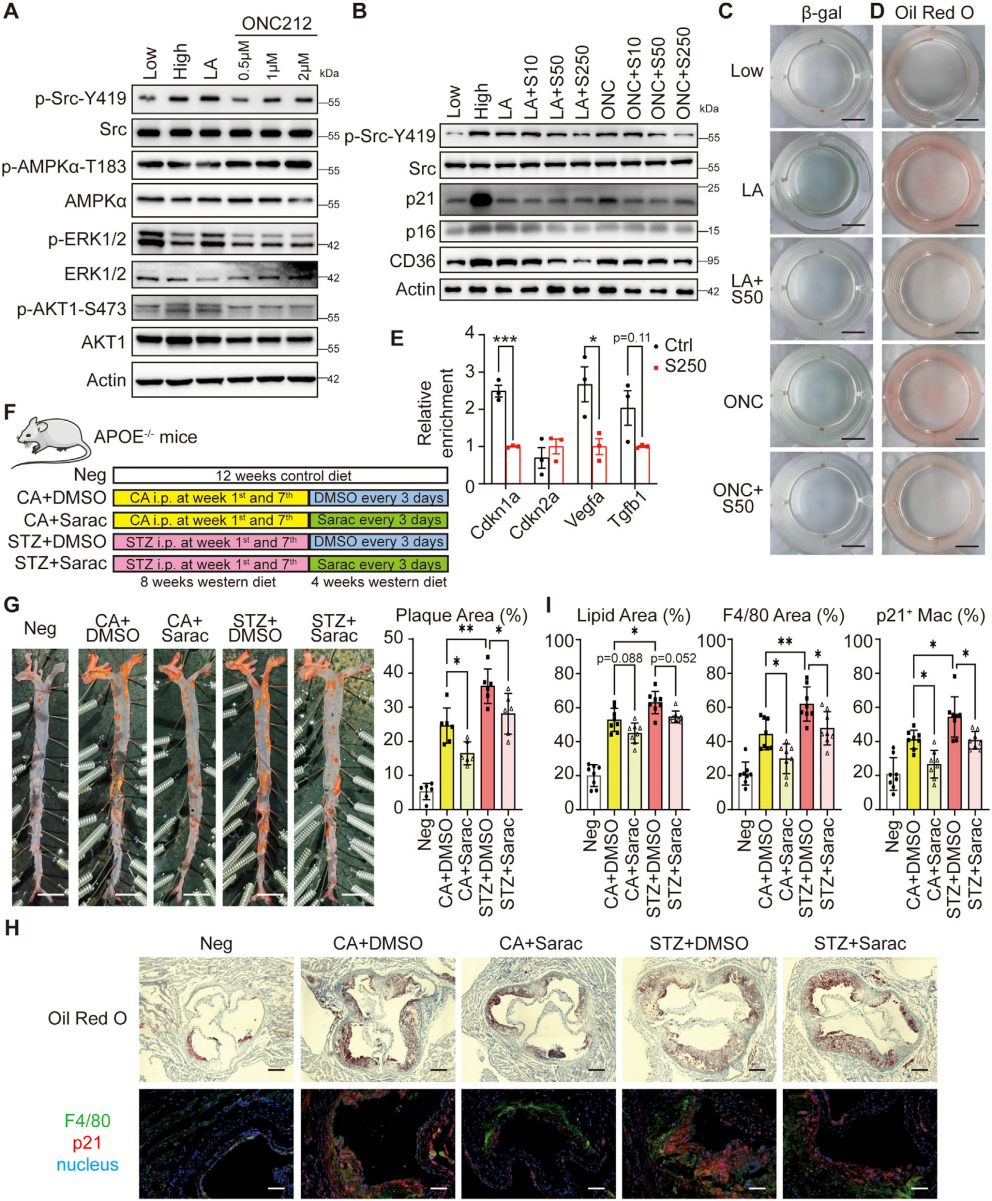
GPR132‐induced Src phosphorylation mediates macrophage senescence and saracatinib is a potential medicine for diabetic atherosclerosis. A) Western blotting to detect the phosphorylation of Src, AMPK, ERK1/2 and AKT1 in the RAW264.7 cells pre‐treated with DMEM with 1.5 mg mL^−1^ glucose (Low), 4.5 mg mL^−1^ glucose (High), 1.5 mg mL^−1^ glucose with 10 mmol/L lactate (LA), 1.5 mg mL^−1^ glucose with different concentration of ONC212 for 24 h pre‐treatment and then 24 h stimulation with 40 mg/mL ox‐LDL. (B) Western blotting to detect the phosphorylation of Src, p16, p21, and CD36 in the RAW264.7 cells with DMEM with 1.5 mg mL^−1^ glucose (Low), 4.5 mg mL^−1^ glucose (High), 1.5 mg mL^−1^ glucose with 10 mmol L^−1^ lactate (LA), 1.5 mg mL^−1^ glucose with 1 µmol L^−1^ of ONC212 (ONC), lactate or ONC212 with different concentration of saracatinib for 24 h pre‐treatment and then 24 hours stimulation with 40 mg mL^−1^ ox‐LDL. S10, S50, S250: saracatinib treatment at 10, 50, and 250 nM concentrations respectively. (C‐D) Representative images of β‐Gal (C) and Oil Red O (D) stained RAW264.7 cells in B. (E) ChIP‐qPCR performed with anti‐STAT3 antibody in RAW264.7 treated by 4.5 mg mL^−1^ glucose with DMSO (Ctrl) or 250 nM saracatinib (S250) for 12 h and then stimulation with 40 mg mL^−1^ ox‐LDL for 12 h. *n* = 3 for each group. (F) A diagram to show the mouse model of diabetic atherosclerosis. The mice were totally fed with the western diet for 12 weeks. 4 mg/kg STZ or citric acid (CA) was injected in the first and seventh weeks of the experiment. From the 9th week, the mice of the experimental groups were treated with DMSO or 20 mg kg^−1^ saracatinib (Sarac) every 3 days for 4 weeks. (G) Representative images of Oil Red O stained aorta. The plaque area is shown in the right panel. *n* = 6 for each group. (H) Representative images of the arterial roots with Oil Red O staining (up), F4/80 (green), and p21 (red) staining (below). (I) Quantification of the plaque area (Oil Red O positive), the macrophage area (F4/80 positive), and the ratio of p21 positive macrophage of the roots. *n* = 8 for each group. The quantitative data in E were analyzed with student t‐tests and the data in G and I were analyzed with one‐way ANOVA. *p < 0.05; **p <0.01; ***p <0.001.

While GPR132 has been implicated in lactate recognition, the downstream signaling pathways remain inadequately defined. We identified several signals of GPCRs and observed that GPR132 activation enhanced phosphorylation of Src (**Figure** **6**A), an oncogene associated with cellular senescence.^[^ ^26^ ^]^ Saracatinib, a specific Src inhibitor, effectively blocked the phosphorylation induced by lactate or ONC212 with a concentration over 50 nM, concurrently suppressing senescence (Figure 6B,C; Figure S6A, Supporting Information). Additionally, the expression of CD36 was downregulated and the uptake of ox‐LDL was diminished (Figure 6B,D; Figure S6B,C, Supporting Information), reinforcing the concept that lactate mediates macrophage senescence and ox‐LDL uptake via the GPR132‐Src signaling pathway. The GPR132‐Src pathway was also confirmed in THP‐1 cells (Figure S6D,E, Supporting Information).

An additional inquiry pertains to the mechanism by which Src regulates macrophage senescence and lipid uptake. Although it is well‐established that Src influences the senescence pathway, the intricate nature of the signaling pathways associated with Src complicates the identification of specific regulatory mechanisms. Notably, a body of literature suggests that Src plays a role in the functional regulation of immune cells through modulation of the STAT3 signaling pathway.^[^ ^27^ ^]^ Moreover, numerous studies have indicated that Src can impact gene expression via the regulation of STAT3.^[^ ^28^ ^]^ Therefore, we performed ChIP experiments utilizing anti‐STAT3 antibodies to elucidate how Src regulates the expression of genes associated with senescence and lipid uptake. As illustrated in Figure 6E, inhibition of Src significantly diminished the binding of STAT3 to the promoters of *Cdkn1a* and *Vegfa*, a finding that is further supported by hTFtarget (https://guolab.wchscu.cn/hTFtarget), suggesting that Src modulates the expression of senescence‐related genes through its influence on STAT3. The binding sites of STAT3 on the promoters of *Cdkn1a* and *Vegfa* could also be found by JASPAR (Figure S7B, Supporting Information). However, it appears that STAT3 does not directly regulate the expression of Cd36 and Olr1 (Figure S7A, Supporting Information), which aligns with our previous data indicating that signaling pathways evolve subsequent to the onset of senescence. To further confirm the transcriptional activity of STAT3 on the senescence signal, a specific inhibitor of STAT3, STAT3‐IN‐3, was employed. Interestingly, STAT3‐IN‐3 effectively abolished the expression of Cdkn1a, Vegfa, and Tgfb1 in a three‐hour inhibition (Figure S7C,D, Supporting Information), showing that STAT3 might be a direct transcript factor of the senescent genes.

To evaluate the potential anti‐senescent and anti‐atherosclerotic effects of saracatinib in vivo, we administered a Western diet along with saracatinib to Apoe^−/−^ mice, both diabetic and non‐diabetic, over a 12‐week period (Figure 6F). Although treatment with saracatinib for four weeks did not result in significant changes in glucose and lipid levels (Figure S8D,E, Supporting Information), it did lead to a reduction of over 30% in plaque burden in both nondiabetic and diabetic mice, alongside a significant decrease in the macrophage content within the plaques (Figure 6H,I). Additionally, a marked reduction in senescent macrophages was noted following saracatinib treatment. Notably, saracatinib also demonstrated an ability to ameliorate atherosclerosis in female mice, regardless of diabetic status (Figure S9A–C, Supporting Information), although the efficacy appeared to be less pronounced compared to male mice. Collectively, these results indicate that targeting macrophage senescence may serve as a viable therapeutic approach for atherosclerosis, independent of lipid levels, with saracatinib emerging as a promising candidate for therapeutic intervention.

### 2.6 Lactate Level in Plasma and GPR132‐Src Activation in PBMCs are Associated with the Severity of Coronary Atherosclerosis in Patients

**Figure 7:**
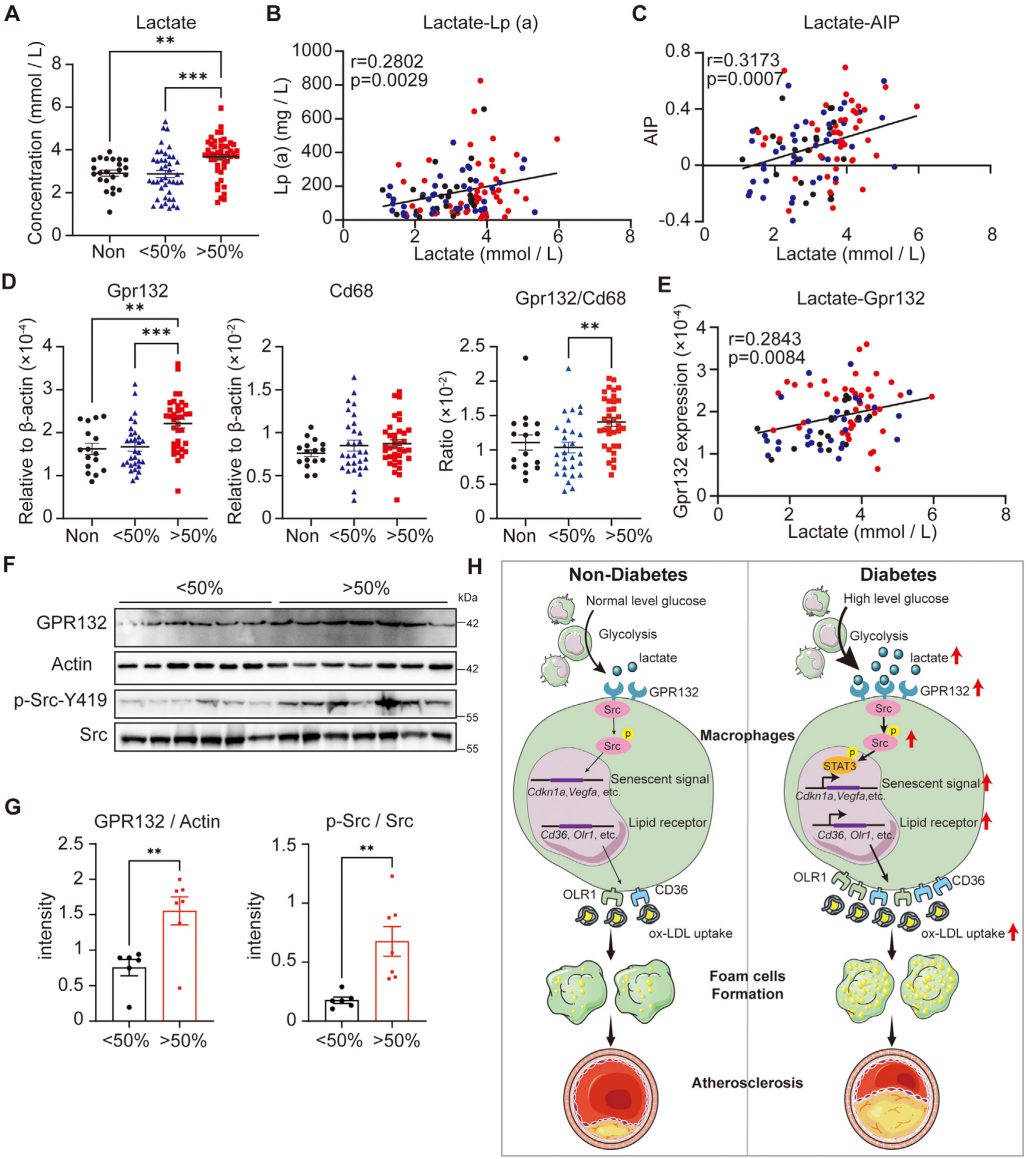
Lactate levels in plasma and GPR132‐Src activation in PBMC are associated with the severity of coronary stenosis in patients. A) Lactate concentration in the plasma of the non‐stenosis patients (Non), stenosis below 50% (<50%), and stenosis over 50% (>50%). Non, *n* = 23; <50%, *n* = 44; >50%, *n* = 48. B, C) The correlation of lactate concentration with Lp (a) level (B) and AIP (C). *n* = 115 for each analysis. D) Relative mRNA levels of Gpr132 and Cd68 in the PBMCs of the patients as in A. Non, *n* = 16; <50%, *n* = 30; >50%, *n* = 36. E) The correlation of lactate concentration with Gpr132 expression. *n* = 82. F) Western blotting to detect GPR132 and the phosphorylation of Src in the PBMCs of the patients as in A. G) Quantification of F. H) A proposed model depicts that lactate‐GPR132‐Src pathway promotes macrophage senescence and foam cell formation to induce atherosclerosis under diabetes. The quantitative data of G were analyzed with student t‐tests, and the quantitative data of A and D were analyzed with one‐way ANOVA. The quantitative data of B, C, E were analyzed with Pearson Correlation. *p < 0.05; **p < 0.01; ***p < 0.001; ****p < 0.0001.

Finally, a comprehensive analysis of blood samples from patients diagnosed with coronary arteriosclerosis was conducted to explore the lactate‐GPR132‐Src signaling pathway. The patient cohort was stratified into three distinct groups based on the severity of coronary stenosis: non‐stenosis (23 patients), stenosis of less than 50% (44 patients), and stenosis exceeding 50% (48 patients). Notably, patients exhibiting more than 50% stenosis demonstrated significantly elevated lactate levels in comparison to the other groups (**Figure** **7**A). Additionally, a positive correlation was established between lactate levels and the atherogenic index of plasma (AIP), while lactate concentration is also potentially associated with LpA (Figure 7B,C).

Moreover, an upregulation of Gpr132 expression was identified in peripheral blood mononuclear cells (PBMCs) from patients with greater than 50% stenosis (Figure 7D). Importantly, the expression of Gpr132 on macrophages, as indicated by the Gpr132/Cd68 ratio, was also significantly elevated. Furthermore, a potential association was observed between lactate concentration and Gpr132 expression (Figure 7E). Western blot analysis corroborated that the GPR132‐Src pathway exhibited heightened activation in patients with more severe stenosis (Figure 7F,G), thereby validating findings from prior animal studies.

## 3 Discussion

Diabetes is recognized as a critical risk factor that exacerbates atherosclerosis, prompting extensive investigation into its underlying mechanisms. Our research proposes a novel hypothesis positing that diabetes may promote macrophage senescence through increased lactate levels, thereby accelerating the progression of atherosclerosis. We provide evidence that elevated glucose concentrations directly induce macrophage senescence, potentially enhancing the uptake of oxidized low‐density lipoprotein (ox‐LDL) by these cells. The increased glucose levels facilitate lactate production via glycolysis, which is consistent with the observed rise in lactate levels in the blood of diabetic patients. Lactate has the capacity to bind to GPR132, activating the Src signaling pathway, which subsequently leads to macrophage senescence and the formation of foam cells (Figure 7H). Clinical samples further substantiate the relationship between lactate levels, GPR132 expression, and the severity of coronary stenosis, highlighting the importance of the lactate‐GPR132‐Src pathway in mediating macrophage senescence in the context of diabetic atherosclerosis. The deletion of Gpr132 has been shown to ameliorate atherosclerosis, while the inhibition of the Src pathway using saracatinib presents a promising therapeutic strategy for addressing diabetic atherosclerosis.

Diabetes is an important risk factor for exacerbating cardiovascular disease (CVD), and in other words, CVDs are the leading cause of morbidity and mortality in individuals with diabetes.^[^ ^29^ ^]^ Effective strategies to reduce the risk of CVD in diabetic patients include lifestyle modifications as a fundamental component, in addition to efforts aimed at optimizing plasma lipid levels, blood pressure, and glycemic control.^[^ ^29^ ^]^ Hyperinsulinemia and dyslipidemia are believed to play critical roles in inflammatory signaling, while elevated glucose levels can alter cholesterol metabolism in monocytes and endothelial cells.^[^ ^30^ ^]^ These mechanisms represent promising therapeutic targets for diabetic atherosclerosis. Intriguingly, antidiabetic interventions have been shown to significantly attenuate vascular calcification, suggesting a strong pathophysiological link between these processes.^[^ ^31^ ^]^ However, the relationship between hyperglycemia and cellular senescence remains poorly understood, highlighting a critical gap in current research. Cellular senescence, particularly within atherosclerotic plaques, is associated with detrimental effects throughout all stages of atherosclerosis.^[^ ^4^ ^]^ Early studies suggest that the senescence of vascular endothelial cells contributes to endothelial dysfunction,^[^ ^11^, ^32^ ^]^ while smooth muscle cell senescence also promotes lesion formation.^[^ ^33^ ^]^ Additionally, increased p16 expression in macrophage‐rich atherosclerotic plaques suggests that macrophages are significant contributors to senescence in developed plaques.^[^ ^11^ ^]^ Various factors have been identified as triggers for cellular senescence, with macrophage senescence appearing to be closely associated with metabolic reprogramming and inflammatory activation, which may accelerate CVDs.^[^ ^34^ ^]^ Our study establishes a connection between diabetes and atherosclerosis through the lens of macrophage senescence, aligning with a series of prior investigations.

Macrophages are multifunctional immune cells that are essential for maintaining tissue homeostasis, defending against pathogens, and regulating inflammation. Their functional plasticity enables them to adapt to diverse microenvironments, largely influenced by metabolic reprogramming.^[^ ^35^ ^]^ Macrophages can polarize into pro‐inflammatory (M1) or anti‐inflammatory (M2) phenotypes, each associated with distinct metabolic pathways. M1 macrophages rely on glycolysis and the pentose phosphate pathway to support rapid energy production and the generation of reactive oxygen species (ROS) and nitric oxide (NO), in contrast, M2 macrophages primarily utilize oxidative phosphorylation and fatty acid oxidation, promoting tissue repair and resolution of inflammation.^[^ ^36^ ^]^ Emerging evidence highlights the importance of metabolic dysregulation in macrophage function during atherosclerosis. For instance, impaired cholesterol efflux pathways and mitochondrial dysfunction in macrophages contribute to foam cell formation and plaque instability.^[^ ^3^, ^37^ ^]^ Targeting macrophage metabolism, such as enhancing fatty acid oxidation or modulating glycolysis, has shown promise in preclinical studies as a strategy to mitigate atherosclerosis and other metabolic diseases.^[^ ^38^ ^]^ Despite the limited number of studies on macrophage senescence, our research suggests a close relationship between cellular senescence and macrophage metabolic disorders. Dysregulation of glucose metabolism can lead to macrophage senescence, which subsequently promotes lipid metabolism dysfunction. Consequently, there exists a complex interplay between the metabolic regulation of macrophages and senescence, raising the important question of whether metabolic interventions in immune cells can directly influence aging processes.

In this study, we performed RNA sequencing of peritoneal macrophages but not aorta macrophages (Figure 1). It is a well‐established fact that most macrophages in arterial plaques originate from peripheral recruitment.^[^ ^39^ ^]^ Peritoneal macrophages share ontogenic and functional similarities with arterial myeloid cells. Both populations originate from common hematopoietic precursors and exhibit conserved inflammatory pathways, such as NLRP3 inflammasome activation and cytokine production.^[^ ^40^ ^]^ On the other hand, peritoneal cells provide a readily accessible and abundant source of immune cells for high‐quality RNA sequencing, whereas arterial plaque‐derived myeloid cells are limited by low yield and technical challenges in isolation, especially in mouse model. There have also been some sequencing cases in the past where peripheral immune cells have been used instead of tissue‐infiltrating immune cells, especially in the field of vascular diseases.^[^ ^41^ ^]^ However, a recent report that macrophages at different sites in the atherosclerosis model exhibit different transcriptomic characteristics.^[^ ^42^ ^]^ Because we have fully confirmed that the macrophage senescence process coexists in peritoneal macrophages and arterial macrophages through the staining of arterial plaques and the downstream related signaling pathways have also been verified by Gpr132 knockout mice and saracatinib‐treatment model, our study also has good confidence. The sequencing of macrophages in vascular situ may be the part that we need to further improve in the future.

Lactate, a significant metabolic intermediate, is acknowledged for its role in the regulation of metabolic homeostasis in aerobic conditions. Prior research has established a correlation between blood lactate concentrations and the risk of diabetes, which may signify the onset of insulin resistance.^[^ ^14^ ^]^ It has been proposed that lactate may inhibit insulin‐mediated glucose uptake while enhancing the pancreatic response to insulin secretagogues.^[^ ^15^ ^]^ Additionally, extensive clinical studies have demonstrated a relationship between blood lactate levels and carotid atherosclerosis,^[^ ^16^ ^]^ implying that elevated lactate concentrations could indicate diminished mitochondrial oxidative capacity, a recognized factor contributing to atherosclerosis. Although earlier studies have characterized lactate as an immunosuppressive agent,^[^ ^43^ ^]^ our findings indicate that lactate promoted macrophage senescence, indicating a novel regulatory role of lactate. Interestingly, a recent study has highlighted lactate's involvement in the generation of immunosenescent B cells,^[^ ^44^ ^]^ validating our hypothesis that lactate is a mediator of senescence. Nevertheless, this hypothesis should be further investigated with more clinical data. Additionally, it is essential to consider the potential adverse effects of lactate accumulation resulting from anaerobic exercise, as recent research has indicated a positive correlation between increased exercise volume and the prevalence of atherosclerotic plaques.^[^ ^45^ ^]^ Of course, in our experiments, we observed that the effect of lactate addition was less pronounced than that of glucose addition (Figure 3), and Gpr132 knockout was found to mitigate, but not completely inhibit, the senescence induced by elevated glucose levels (Figure 5). These results indicates that the lactate‐GPR132‐Src signaling pathway is not the sole mechanism through which a hyperglycemic environment induces cellular senescence. In addition, intracellular lactate plays a pivotal role in macrophage regulation. Recent studies have demonstrated that lactate‐mediated epigenetic modifications can suppress macrophage‐driven inflammation, thereby ameliorating atherosclerosis.^[^ ^46^ ^]^ Given that the progression of atherosclerosis is governed by complex epigenetic mechanisms,^[^ ^47^ ^]^ it is plausible that lactate‐induced modifications significantly influence disease pathogenesis. Further investigation into this relationship will be a critical focus of future research. Furthermore, additional diabetes‐associated metabolites such as citrate, succinate, and glutamate may contribute to atherosclerotic pathogenesis.^[^ ^48^ ^]^ Our preliminary findings suggest that certain metabolites participate in endothelial dysfunction and macrophage senescence, the detailed mechanisms of which will be elucidated in our future research.

GPR132, also known as G2A, exhibits high expression levels in macrophages, where lactate serves as a high‐affinity ligand.^[^ ^25b^ ^]^ We note that the expression of HCAR1 and GPR132 appears to be elevated in the presence of lactate supplementation (Figure 5D), suggesting that both may indeed be regulated by feedback signals of lactate. In addition, we chose GPR132 over the more classical lactate receptor HCAR1 as the subject of study mainly due to the agonist experiments (Figure 5E), which point out that GPR132 may be more important in the senescence process. Previous studies have documented that GPR132 modulates various cellular biological processes.^[^ ^49^ ^]^ Notably, it has been reported that GPR132 can inhibit the cell cycle in response to stress,^[^ ^50^ ^]^ demonstrating its regulatory function on cellular senescence. Prior to our research, it was established that GPR132 influences the progression of atherosclerosis in Ldlr^−/−^ mice,^[^ ^51^ ^]^ which aligns with our findings. Furthermore, recent evidence indicates that macrophage‐specific knockout or inhibition of GPR132 enhances glucose homeostasis,^[^ ^52^ ^]^ indicating that GPR132 may be a critical target for metabolic diseases. The downstream Src kinase of GPCRs is a signaling determinant of cellular senescence,^[^ ^26^ ^]^ which also plays a complicated role in the pathogenesis of CVD.^[^ ^53^ ^]^ Our study supports that saracatinib can mitigate atherosclerosis by inhibiting macrophage senescence rather than by reducing blood lipid levels, underscoring the role of Src‐mediated cellular senescence in atherosclerosis. Interestingly, recent studies have identified saracatinib as an effective therapeutic agent for atherosclerosis through metabolic reprogramming in peripheral macrophages and DCs.^[^ ^54^ ^]^ Given the critical role of metabolic disorders in cellular senescence, our results are in alignment with this body of research. And it is very valuable that we note that the administration of saracatinib did not appear to be associated with significant side effects in either in vitro or in vivo settings (Figure S10, Supporting Information), highlighting the potential for clinical translation of this drug. In conclusion, targeting GPR132 or Src may present promising therapeutic strategies in conjunction with lipid‐lowering agents. Of course, though our genetic approach using Gpr132^−/−^ BMDMs (Figure 5) provides compelling preliminary evidence for GPR132's involvement in macrophage senescence, we agree that future studies with specific GPR132 inhibitors would be valuable to detect the signal in vitro and in vivo.

We also verified that STAT3 might be the downstream signaling induced by Src to regulate the expression of senescent genes by bioinformatic analysis, ChIP‐qPCR, and pharmacological inhibition (Figure 6E; Figure S7, Supporting Information). However, we observed that acute STAT3 inhibition effectively blocked glucose‐induced expression of Cdkn1a and Vegfa but prolonged inhibition either lost efficacy or paradoxically enhanced expression of these genes. We speculate this time‐dependent effect may reflect the dual nature of STAT3 signaling, which is known to regulate proliferative pathways in cancers. The complex temporal dynamics observed suggest that while Src likely modulates senescence signals through STAT3, additional regulatory mechanisms may become prominent during sustained inhibition.

Our research does have certain limitations. First, lactate appears to partially elucidate the regulation of macrophage senescence in a high‐glucose environment, which involves increasingly complex mediators that have not been thoroughly investigated. Second, flow cytometry or single‐cell sequencing of macrophages in plaques may be a more accurate and excellent protocol, but due to technical limitations, it was not performed in this study. Thirdly, whether the lactate‐GPR132‐Src axis represents a generalized mechanism for atherosclerotic plaque formation throughout the vasculature needs further exploration. Finally, lactate concentration in humans shows a positive but weak association with AIP and LpA, so a more reliable study may need to further expand the sample size. We anticipate that further studies will address these questions and evaluate the efficacy of targeting inhibitors in mouse models and potentially in human subjects in the future.

## 4 Experimental Section

### Human Samples

The analysis presented in Figure 1 encompasses data from 297 hospitalized patients within the cardiology division‐5 of Xiamen Cardiovascular Hospital, collected in January 2024. A total of 297 patients underwent screening, with 266 patients meeting the inclusion criteria (Figure 1A). Detailed information regarding the characteristics of the patient cohorts is available in Table S1 (Supporting Information). The category labeled “Others” comprises patients diagnosed with heart failure, myocardial infarction, and various other conditions, excluding those with atherosclerosis. Patients exhibiting abnormal liver and kidney function were excluded from the study. The enrolled patients were stratified into three distinct groups based on HbA1c levels or glucose concentrations, in accordance with the guidelines established by the International Diabetes Federation (IDF) and the American Diabetes Association (ADA):^[^ ^55^ ^]^ individuals with random venous plasma glucose levels ≥ 11.1 mmol L^−1^ or HbA1c ≥ 6.5% were classified as having diabetes; those with glucose levels ≥ 7.8 mmol L^−1^ or HbA1c ranging from 5.8% to 6.5% were categorized as pre‐diabetic; and the remaining individuals were considered to have normal glucose metabolism. A total of 142 patients with recorded HbA1c levels were included for correlation analysis.

In Figure 6, data from 115 hospitalized patients, both with and without coronary arteriosclerosis, were collected following an overnight fast using heparinized tubes. The relevant characteristics of these individuals are detailed in Table S2 (Supporting Information). Exclusion criteria for this cohort included abnormal liver and kidney function, stage three hypertension, and any history of biguanide medication within the prior week. Plasma samples were obtained through centrifugation at 4 °C for 10 min at 1000×g, while peripheral blood mononuclear cells (PBMCs) were isolated using density gradient centrifugation.

All sampling procedures adhered to the ethical principles outlined in the Declaration of Helsinki and received approval from the Ethics Committee of Xiamen Cardiovascular Hospital (2024‐YLK‐33). Informed written consent was obtained from each participant prior to their inclusion in the study.

### Animal Studies

The Apoe^−/−^ mice and Gpr132^−/−^ mice were purchased from GemPharmatech (Nanjing, China). All the mice were on C57/BL6J background. Animals were maintained and used in accordance with the guidelines of the Institutional Animal Care and Use Committee of Xiamen University (approval no. XMULAC20230163). The mice were maintained under a 12‐h light/dark cycle at a temperature of 25 °C. Both male and female mice were utilized for the experiments.

The mice were randomly allocated to experimental groups and induced to develop diabetes when their body weight exceeded 25 g. This was achieved through intraperitoneal administration of streptozotocin (STZ) at a dosage of 40 mg kg^−1^ over 5 consecutive days during the first week, followed by an additional 2 consecutive days of treatment during either the 5th or 7th week. Mice exhibiting fasting blood glucose levels exceeding 12.5 mmol L^−1^ by the 4th‐week post‐STZ treatment were selected for further study. Following the initiation of STZ injections, the mice were placed on a Western diet, which comprised 40% caloric intake from fat and included an additional 1.25% cholesterol (Ready Dietech D12108C). For the administration of saracatinib, the mice received either 20 mg kg^−1^ of saracatinib or DMSO via oral gavage every three days for a duration of four weeks.

To assess glucose levels, the mice were subjected to a 6‐h fasting period, after which blood samples were collected from the tail tips. For the measurement of triglycerides (TG), total cholesterol (TC), high‐density lipoprotein cholesterol (HDL‐C), and low‐density lipoprotein cholesterol (LDL‐C), a similar fasting protocol was followed, with blood samples obtained from the canthus under deep anesthesia. For aortic staining, cardiac perfusion with phosphate‐buffered saline (PBS) was performed immediately following euthanasia. The aorta was then carefully dissected and fixed, with any attached fat and external adventitia removed prior to staining. For Oil Red O staining or β‐Gal staining, the aorta was dissected at the median membrane and stained according to the manufacturer's instructions provided by Beyotime, Shanghai (C0157 or C0602). Investigators conducting histological and biochemical analyses were blinded to the treatment groups to ensure objectivity.

### Cell Culture and Treatment

RAW264.7 cells and THP‐1 cells were gifts from Dr. Jun Wu, Xiamen Cardiovascular Hospital. RAW264.7 cells were cultured in 1.5 g L^−1^ glucose DMEM with 100 units mL^−1^ penicillin/ streptomycin and 10% FBS at 37 °C with 5% CO_2_. For treatment, RAW264.7 cells were seeded in plates overnight and then cultured with DMEM containing different levels of glucose for 12 h with other compounds. After that, 40 mg mL^−1^ ox‐LDL (Guangzhou Yiyuan Biotech. Co. Ltd, YB‐002) or 10 mg mL^−1^ Dil‐ox‐LDL (YB‐0010) was added for 24 h. THP‐1 cells were cultured in RPMI1640 with 100 units mL^−1^ penicillin/ streptomycin and 10% FBS at 37 °C with 5% CO_2_. For treatment, THP‐1 cells were seeded in plates overnight with 100 ng mL^−1^ Phorbol 12‐myristate 13‐acetate (PMA), and then cultured with DMEM containing different levels of glucose for 12 h with other compounds, followed by 50 mg mL^−1^ ox‐LDL addition for 24 h. Bone marrow‐derived macrophages (BMDMs) isolation was carried out as described.^[^ ^56^ ^]^ For Oil Red O or β‐Gal staining, cells were fixed and stained following the instructions of the kit described above.

### Reagents

All primary antibodies and drugs are shown in Table S3 (Supporting information). The HRP‐conjugated secondary antibodies were from ABclonal (1:3000, AS014, and AS003). Alexa Fluor 488/546 conjugated secondary antibodies were from Invitrogen (1:100, A11001, and A11030).

### Immunohistochemistry and Immunofluorescent Staining

Mouse hearts were cut stick to the root of the aorta and fixed with 4% PFA for 24 h for OCT‐embedding. Hematoxylin‐eosin staining and IHC were carried out as described and imaged with EVOS FL AUTO2 microscope.^[^ ^56^ ^]^ For immunofluorescent staining, sections were washed with 0.1% Triton X100 in PBS after returning to room temperature and blocked with goat serum for 1 h, then incubated with primary and secondary antibodies for 2 h respectively. The nuclei were stained with Hoechst33342. Fluorescence images were acquired with Leica Stellaris 5.

The staining area was quantified with ImageJ. Open the picture with ImageJ, and manually frame the areas of arteries and plaques, then use the “Analyze‐Measure” option of the software to calculate the area.

### RNA Isolation, RNA Sequencing and RT‐qPCR

Cells were lysed by TRIzol reagent (Invitrogen, CA) and RNA was purified according to the manufacturer's instructions. RNA libraries were prepared with a SMARTER mRNA‐seq library kit, and sequencing was performed by Novogene (Beijing, China).

For qPCR, RNA was reverse‐transcribed with Hifair AdvanceFast 1st Strand cDNA Synthesis Kit (Yeasen, Shanghai, China) to obtain cDNA. Real‐time PCR was performed with Genious SYBR Green Fast qPCR Mix (Abclonal, Shanghai, China).

### Protein Extraction and Immunoblotting

Cells were lysed in RIPA buffer with complete protease inhibitors and phosphatase inhibitors (Roche), and the supernatant was collected after centrifugation at 4 °C for 10 min, 12000 rpm. The proteins were eluted with SDS sample buffer (LABLEAD, Beijing, China) and assayed by western blotting using 10%–12% SDS‐PAGE gels.

### Chromatin Immunoprecipitation qPCR (ChIP‐qPCR)

The ChIP‐qPCR was carried out as described.^[^ ^56^ ^]^ RAW264.7 cells were cultured in DMEM with 4.5 g L^−1^ glucose with DMSO or 250 nM saracatinib for 24 h, and then fixed with 1% (wt/vol) formaldehyde for 10 min, subsequently followed by quenching with glycine. The cells were then resuspended with lysis buffer and washed twice respectively with washing buffer and shearing buffer to collect the nucleus. The chromatin was sonicated to generate 200‐ to 1000‐bp fragments with Bioruptor (Diagenode minichiller 300). Then the supernatant was collected and washed, incubated with dynabeads for pre‐clearance, with 5% as input and other incubating with anti‐STAT3‐coated beads overnight. The beads were washed with high salt buffer and TE, followed by elution of DNA and de‐crosslinking. DNA was purified with DNA concentration kit (Tiangen, Beijing, China). qPCR data of each sample was normalized with input.

### Statistics

All data were shown as mean ± SEM. All data from clinical detection and animal experiments were filtered by average±2S.D. (95% confidence interval). Statistical analyses were performed using Graph Prism 9.5 software. Quantitation results were analyzed as figure legend introduction. Values of *p* < 0.05 were considered statistically significant.

## Conflict of Interest

The authors have declared that no conflict of interest exists.

## Author Contributions

Y.L. and C.D. designed the experiments. X.G., Z.L., and J.Y. performed clinical data collection and analysis. S.W. and Y.L. performed the animal and cellular experiments and data analysis. Y.L. wrote the manuscript and prepared the figures. B.L., R.W., S.B., and N. provided assistance for experiments. Y.W. and C.D. provided comments, supervised the study, and provided writing revision. All authors read and approved the manuscript. Y.L. is the guarantor of this work and takes responsibility for the integrity of the data and the accuracy of the data analysis. X.G., S.W., Z.L., and J.Y. contributed equally to this study.

## Supporting information



Supporting Information

## Data Availability

The data that support the findings of this study are available from the corresponding author upon reasonable request.

## References

[1] P. Libby , Nature 2021, 592, 524. 33883728 10.1038/s41586-021-03392-8

[2] J. L. M. Bjorkegren , A. J. Lusis , Cell 2022, 185, 1630. 35504280 10.1016/j.cell.2022.04.004 PMC9119695

[3] I. Tabas , K. E. Bornfeldt , Circ. Res. 2016, 118, 653. 26892964 10.1161/CIRCRESAHA.115.306256 PMC4762068

[4] B. G. Childs , D. J. Baker , T. Wijshake , C. A. Conover , J. Campisi , J. M. van Deursen , Science 2016, 354, 472. 27789842 10.1126/science.aaf6659 PMC5112585

[5] a) M. L. Chao , S. Luo , C. Zhang , X. Zhou , M. Zhou , J. Wang , C. Kong , J. Chen , Z. Lin , X. Tang , S. Sun , X. Tang , H. Chen , H. Wang , D. Wang , J. P. Sun , Y. Han , L. Xie , Y. Ji , Nat. Commun. 2021, 12, 4452; 34294713 10.1038/s41467-021-24736-y PMC8298471

[6] C. B. Renard , F. Kramer , F. Johansson , N. Lamharzi , L. R. Tannock , M. G. von Herrath , A. Chait , K. E. Bornfeldt , J. Clin. Invest. 2004, 114, 659. 15343384 10.1172/JCI17867 PMC514580

[7] a) C. E. Tabit , S. M. Shenouda , M. Holbrook , J. L. Fetterman , S. Kiani , A. A. Frame , M. A. Kluge , A. Held , M. M. Dohadwala , N. Gokce , M. G. Farb , J. Rosenzweig , N. Ruderman , J. A. Vita , N. M. Hamburg , Circulation 2013, 127, 86; 23204109 10.1161/CIRCULATIONAHA.112.127514 PMC3572725

[8] a) S. Das , M. A. Reddy , P. Senapati , K. Stapleton , L. Lanting , M. Wang , V. Amaram , R. Ganguly , L. Zhang , S. Devaraj , D. E. Schones , R. Natarajan , Arterioscler Thromb. Vasc. Biol. 2018, 38, 1806; 29930005 10.1161/ATVBAHA.117.310663 PMC6202204

[9] S. P. Gray , E. Di Marco , J. Okabe , C. Szyndralewiez , F. Heitz , A. C. Montezano , J. B. de Haan , C. Koulis , A. El‐Osta , K. L. Andrews , J. P. Chin‐Dusting , R. M. Touyz , K. Wingler , M. E. Cooper , H. H. Schmidt , K. A. Jandeleit‐Dahm , Circulation 2013, 127, 1888. 23564668 10.1161/CIRCULATIONAHA.112.132159

[10] C. Lopez‐Otin , M. A. Blasco , L. Partridge , M. Serrano , G. Kroemer , Cell 2023, 186, 243. 36599349 10.1016/j.cell.2022.11.001

[11] L. M. Holdt , K. Sass , G. Gabel , H. Bergert , J. Thiery , D. Teupser , Atherosclerosis 2011, 214, 264. 20637465 10.1016/j.atherosclerosis.2010.06.029

[12] A. K. Palmer , B. Gustafson , J. L. Kirkland , U. Smith , Diabetologia 2019, 62, 1835. 31451866 10.1007/s00125-019-4934-x PMC6731336

[13] a) Y. Lin , M. Bai , S. Wang , L. Chen , Z. Li , C. Li , P. Cao , Y. Chen , Diabetes 2022, 71, 637; 35044451 10.2337/db21-0535

[14] Y. D. Chen , B. B. Varasteh , G. M. Reaven , Diabete Metab. 1993, 19, 348. 8293860

[15] Y. Wu , Y. Dong , M. Atefi , Y. Liu , Y. Elshimali , J. V. Vadgama , Mediators Inflamm. 2016, 6456018. 28077918 10.1155/2016/6456018 PMC5203906

[16] G. P. Shantha , B. Wasserman , B. C. Astor , J. Coresh , F. Brancati , A. R. Sharrett , J. H. Young , Atherosclerosis 2013, 228, 249. 23510829 10.1016/j.atherosclerosis.2013.02.014 PMC3657708

[17] S. B. Uzunget , K. E. Sahin , BMC Cardiovasc. Disord. 2022, 22, 511. 36451082 10.1186/s12872-022-02891-4 PMC9710030

[18] J. Sanada , T. Kimura , M. Shimoda , Y. Iwamoto , H. Iwamoto , K. Dan , Y. Fushimi , Y. Katakura , Y. Nogami , Y. Shirakiya , Y. Yamasaki , T. Ikeda , S. Nakanishi , T. Mune , K. Kaku , H. Kaneto , Cardiovasc. Diabetol. 2024, 23, 105. 38504316 10.1186/s12933-024-02189-z PMC10953273

[19] K. J. Moore , F. J. Sheedy , E. A. Fisher , Nat. Rev. Immunol. 2013, 13, 709. 23995626 10.1038/nri3520 PMC4357520

[20] K. Tighanimine , J. A. Nabuco Leva Ferreira Freitas , I. Nemazanyy , A. Bankole , D. Benarroch‐Popivker , S. Brodesser , G. Dore , L. Robinson , P. Benit , S. Ladraa , Y. B. Saada , B. Friguet , P. Bertolino , D. Bernard , G. Canaud , P. Rustin , E. Gilson , O. Bischof , S. Fumagalli , M. Pende , Nat. Metab. 2024, 6, 323. 38409325 10.1038/s42255-023-00972-y PMC10896726

[21] a) M. Chong , T. Yin , R. Chen , H. Xiang , L. Yuan , Y. Ding , C. C. Pan , Z. Tang , P. B. Alexander , Q. J. Li , X. F. Wang , EMBO Rep. 2018, 19, 45274; 10.15252/embr.201745274 PMC5989758 29777051

[22] X. Dou , Q. Fu , Q. Long , S. Liu , Y. Zou , D. Fu , Q. Xu , Z. Jiang , X. Ren , G. Zhang , X. Wei , Q. Li , J. Campisi , Y. Zhao , Y. Sun , Nat. Metab. 2023, 5, 1887. 37903887 10.1038/s42255-023-00912-w PMC10663165

[23] A. Kumar , P. Gupta , M. Rana , T. Chandra , M. Dikshit , M. K. Barthwal , J. Lipid Res. 2020, 61, 351. 31988148 10.1194/jlr.RA119000382 PMC7053835

[24] L. Ippolito , A. Morandi , E. Giannoni , P. Chiarugi , Trends Biochem. Sci. 2019, 44, 153. 30473428 10.1016/j.tibs.2018.10.011

[25] a) K. Ahmed , S. Tunaru , C. Tang , M. Muller , A. Gille , A. Sassmann , J. Hanson , S. Offermanns , Cell Metab. 2010, 11, 311; 20374963 10.1016/j.cmet.2010.02.012

[26] a) X. Xiao , M. Xu , H. Yu , L. Wang , X. Li , J. Rak , S. Wang , R. C. Zhao , Signal Transduct. Targeted. Ther. 2021, 6, 354; 10.1038/s41392-021-00765-3 PMC8531331 34675187

[27] a) H. Yu , D. Pardoll , R. Jove , Nat. Rev. Cancer 2009, 9, 798; 19851315 10.1038/nrc2734 PMC4856025

[28] a) C. L. Yu , D. J. Meyer , G. S. Campbell , A. C. Larner , C. Carter‐Su , J. Schwartz , R. Jove , Science 1995, 269, 81; 7541555 10.1126/science.7541555

[29] N. D. Wong , N. Sattar , Nat. Rev. Cardiol. 2023, 20, 685. 37193856 10.1038/s41569-023-00877-z

[30] T. Hayek , M. Kaplan , R. Kerry , M. Aviram , Atherosclerosis 2007, 195, 277. 17258748 10.1016/j.atherosclerosis.2006.12.026

[31] S. Ghosh , D. Luo , W. He , J. Chen , X. Su , H. Huang , Pharmacol. Res. 2020, 158, 104861. 32407954 10.1016/j.phrs.2020.104861

[32] T. Minamino , I. Komuro , Circ. Res. 2007, 100, 15. 17204661 10.1161/01.RES.0000256837.40544.4a

[33] a) J. Wang , A. K. Uryga , J. Reinhold , N. Figg , L. Baker , A. Finigan , K. Gray , S. Kumar , M. Clarke , M. Bennett , Circulation 2015, 132, 1909; 26416809 10.1161/CIRCULATIONAHA.115.016457

[34] P. S. Minhas , A. Latif‐Hernandez , M. R. McReynolds , A. S. Durairaj , Q. Wang , A. Rubin , A. U. Joshi , J. Q. He , E. Gauba , L. Liu , C. Wang , M. Linde , Y. Sugiura , P. K. Moon , R. Majeti , M. Suematsu , D. Mochly‐Rosen , I. L. Weissman , F. M. Longo , J. D. Rabinowitz , K. I. Andreasson , Nature 2021, 590, 122. 33473210 10.1038/s41586-020-03160-0 PMC8274816

[35] P. J. Murray , T. A. Wynn , Nat. Rev. Immunol. 2011, 11, 723. 21997792 10.1038/nri3073 PMC3422549

[36] L. A. O'Neill , E. J. Pearce , J. Exp. Med. 2016, 213, 15. 26694970 10.1084/jem.20151570 PMC4710204

[37] M. I. Cybulsky , C. Cheong , C. S. Robbins , Circ. Res. 2016, 118, 637. 26892963 10.1161/CIRCRESAHA.115.306542

[38] a) D. A. Jaitin , L. Adlung , C. A. Thaiss , A. Weiner , B. Li , H. Descamps , P. Lundgren , C. Bleriot , Z. Liu , A. Deczkowska , H. Keren‐Shaul , E. David , N. Zmora , S. M. Eldar , N. Lubezky , O. Shibolet , D. A. Hill , M. A. Lazar , M. Colonna , F. Ginhoux , H. Shapiro , E. Elinav , I. Amit , Cell 2019, 178, 686; 31257031 10.1016/j.cell.2019.05.054 PMC7068689

[39] F. K. Swirski , M. J. Pittet , M. F. Kircher , E. Aikawa , F. A. Jaffer , P. Libby , R. Weissleder , Proc. Natl. Acad. Sci. U.S.A. 2006, 103, 10340. 16801531 10.1073/pnas.0604260103 PMC1502459

[40] a) S. Yona , K. W. Kim , Y. Wolf , A. Mildner , D. Varol , M. Breker , D. Strauss‐Ayali , S. Viukov , M. Guilliams , A. Misharin , D. A. Hume , H. Perlman , B. Malissen , E. Zelzer , S. Jung , Immunity 2013, 38, 79; 23273845 10.1016/j.immuni.2012.12.001 PMC3908543

[41] a) Y. Liu , Y. Zhong , H. Chen , D. Wang , M. Wang , J. S. Ou , M. Xia , Circulation 2017, 135, 1339; 28122883 10.1161/CIRCULATIONAHA.116.024503

[42] C. Hardtner , A. Kumar , C. A. Ehlert , T. A. Vico , C. Starz , A. von Ehr , K. Krebs , B. Dufner , N. Hoppe , P. Stachon , T. Heidt , D. Wolf , C. von Zur Muhlen , B. Gruning , C. S. Robbins , L. Maegdefessel , D. Westermann , T. S. Dederichs , I. Hilgendorf , Atherosclerosis 2023, 371, 1. 36940535 10.1016/j.atherosclerosis.2023.03.006

[43] D. Zhang , Z. Tang , H. Huang , G. Zhou , C. Cui , Y. Weng , W. Liu , S. Kim , S. Lee , M. Perez‐Neut , J. Ding , D. Czyz , R. Hu , Z. Ye , M. He , Y. G. Zheng , H. A. Shuman , L. Dai , B. Ren , R. G. Roeder , L. Becker , Y. Zhao , Nature 2019, 574, 575. 31645732 10.1038/s41586-019-1678-1 PMC6818755

[44] M. Romero , K. Miller , A. Gelsomini , D. Garcia , K. Li , D. Suresh , D. Frasca , Nat. Commun. 2024, 15, 7515. 39209820 10.1038/s41467-024-51207-x PMC11362567

[45] a) V. L. Aengevaeren , A. Mosterd , T. L. Braber , N. H. J. Prakken , P. A. Doevendans , D. E. Grobbee , P. D. Thompson , T. M. H. Eijsvogels , B. K. Velthuis , Circulation 2017, 136, 138; 28450347 10.1161/CIRCULATIONAHA.117.027834

[46] L. Chen , M. Zhang , X. Yang , Y. Wang , T. Huang , X. Li , Y. Ban , Q. Li , Q. Yang , Y. Zhang , Y. Zheng , D. Wang , X. Wang , X. Shi , M. Zhang , Y. Sun , J. Wu , Theranostics 2024, 14, 4256. 39113793 10.7150/thno.94738 PMC11303070

[47] Y. Shi , H. Zhang , S. Huang , L. Yin , F. Wang , P. Luo , H. Huang , Signal Transduct. Target Ther. 2022, 7, 200. 35752619 10.1038/s41392-022-01055-2 PMC9233709

[48] Z. Zhou , B. Dong , D. He , J. Ma , Y. Kong , H. Zhu , C. Xie , T. Yang , X. Zhen , Z. Zhang , Z. He , J. Cheng , A. Huang , J. Chen , R. Wu , H. Yin , Y. Chen , J. Tao , H. Huang , Adv. Sci. 2025, 2414252. 10.1002/advs.202414252 PMC12140355 40289670

[49] a) L. Q. Le , J. H. Kabarowski , Z. Weng , A. B. Satterthwaite , E. T. Harvill , E. R. Jensen , J. F. Miller , O. N. Witte , Immunity 2001, 14, 561; 11371358 10.1016/s1074-7613(01)00145-5

[50] Z. Weng , A. C. Fluckiger , S. Nisitani , M. I. Wahl , L. Q. Le , C. A. Hunter , A. A. Fernal , M. M. Le Beau , O. N. Witte , Proc. Natl. Acad. Sci. U.S.A. 1998, 95, 12334. 9770487 10.1073/pnas.95.21.12334 PMC22832

[51] B. W. Parks , A. J. Lusis , J. H. Kabarowski , Arterioscler. Thromb. Vasc. Biol. 2006, 26, 2703. 16990555 10.1161/01.ATV.0000246774.02426.71

[52] J. L. Wang , X. D. Dou , J. Cheng , M. X. Gao , G. F. Xu , W. Ding , J. H. Ding , Y. Li , S. H. Wang , Z. W. Ji , X. Y. Zhao , T. Y. Huo , C. F. Zhang , Y. M. Liu , X. Y. Sha , J. R. Gao , W. H. Zhang , Y. Hao , C. Zhang , J. P. Sun , N. Jiao , X. Yu , Nat. Metab. 2023, 5, 1726. 37770763 10.1038/s42255-023-00899-4

[53] M. Hussain , W. Ikram , U. Ikram , Mol. Genet. Genomics 2023, 298, 315. 36700976 10.1007/s00438-023-01992-9

[54] L. Amadori , C. Calcagno , D. M. Fernandez , S. Koplev , N. Fernandez , R. Kaur , P. Mury , N. S. Khan , S. Sajja , R. Shamailova , Y. Cyr , M. Jeon , C. A. Hill , P. S. Chong , S. Naidu , K. Sakurai , A. A. Ghotbi , R. Soler , N. Eberhardt , A. Rahman , P. Faries , K. J. Moore , Z. A. Fayad , A. Ma'ayan , C. Giannarelli , Nat. Cardiovasc. Res. 2023, 2, 550. 37771373 10.1038/s44161-023-00278-y PMC10538622

[55] a) P. Aschner , S. Karuranga , S. James , D. Simmons , A. Basit , J. E. Shaw , S. H. Wild , K. Ogurtsova , P. Saeedi , Diabetes Res Clin Pract 2021, 172, 108630; 33347900 10.1016/j.diabres.2020.108630

[56] Y. Lin , M. Huang , S. Wang , X. You , L. Zhang , Y. Chen , Immunology 2021, 163, 60. 33421113 10.1111/imm.13303 PMC8044334

