# Supplementary material for: Lactate‐Activated GPR132‐Src Signal Induces Macrophage Senescence and Aggravates Atherosclerosis Under Diabetes
Source: Adv Sci (Weinh). 2025 Jun 10;12(33):e00141. doi: 10.1002/advs.202500141 (PMC12412570; doi:10.1002/advs.202500141)
Supplement: Supplementary file 1 — Supporting Information [file ADVS-12-e00141-s001.docx]

| Supplementary Table 1. Characteristics of human subjects in Figure 1 | | | |
| --- | --- | --- | --- |
| Characteristics | **Coronary arteriosclerosis (n=169)** | **Other (n=97)** | **p Value** |
| Male (%) | 112 (66.3) | 60 (61.9) | 0.4683 |
| Age (years) | 67.39 ± 11.76 | 65.05 ± 13.65 | 0.1426 |
| HbA1c, % | 6.448 ± 1.096 | 6.076 ± 1.346 | 0.0181 |
| Diabetes or Pre-diabetes (%) | 129 (76.3) | 55 (56.7) | 0.0008 |
| ALT, U/L | 21.08 ± 14.59 | 20.50 ± 13.03 | 0.7444 |
| AST, U/L | 25.66 ± 13.64 | 24.06 ± 13.22 | 0.3512 |
| TC, mmol/L | 4.186 ± 1.658 | 3.424 ± 2.154 | 0.001492 |
| TG, mmol/L | 2.224 ± 5.201 | 1.703 ± 0.8808 | 0.3255 |
| HDL-C, mmol/L | 1.077 ± 0.2616 | 1.176 ± 0.4467 | 0.02515 |
| LDL-C, mmol/L | 2.773 ± 1.015 | 2.188 ± 1.251 | 0.006952 |
| APOA1, g/L | 1.177 ± 0.2341 | 1.284 ± 0.4122 | 0.007934 |
| APOB, g/L | 1.028 ± 0.2685 | 0.9424 ± 0.2584 | 0.01214 |
| AIP | 0.1732 ± 0.2894 | 0.1206 ± 0.2510 | 0.1470 |
|  |  |  |  |
| All human subjects were recruited in the Xiamen Cardiovascular Hospital of Xiamen University in January, 2024. All human subjects were Han Chinese. Data are expressed as Mean ± SD. Chi-square test for Sex and Diabetes; Two-tailed unpaired student's t-test for other indexes.  Abbrevations: HbA1c, Glycosylated hemoglobin; ALT, alanine aminotransferase; AST, aspartate aminotransferase; TC, total cholesterol; TG, triglyceride; HDL-C, high-density lipoprotein cholesterol; LDL-C, low-density lipoprotein cholesterol; APOA1, apolipoprotein A1; APOB, apolipoprotein B; AIP, atherosclerotic index of plasma. | | | |
|  | | | |

| Supplementary Table 2. Characteristics of human subjects in Figure 7 | | | |
| --- | --- | --- | --- |
| Characteristics | **Non-stenosis (n=23)** | **Stenosis<50% (n=44)** | **Stenosis>50% (n=48)** |
| Male (%) | 12 (52.2) | 22 (50) | 36 (75) |
| Age (years) | 52.57 ± 11.76 | 65.44 ± 10.66 | 63.98 ± 11.01 |
| AIP | 0.1396 ± 0.2559 | 0.1182 ± 0.2350 | 0.1815 ± 0.2638 |
| Diabetes/Pre/Non | 3/3/17 | 7/10/27 | 7/8/19 |
| Hypertension(Non/I/II) | 16/5/2 | 27/8/9 | 22/14/12 |
| FBG, mmol/L | 5.194 ± 1.265 | 5.369 ± 1.148 | 5.026 ± 1.203 |
| LA, mmol/L | 2.799 ± 0.7242 | 2.883 ± 1.031 | 3.667 ± 0.9071 |
| TC, mmol/L | 4.515 ± 0.6666 | 4.168 ± 1.002 | 3.974 ±1.101 |
| TG, mmol/L | 1.801 ± 1.186 | 1.524 ± 0.7090 | 1.543 ± 0.7365 |
| HDL-C, mmol/L | 1.150 ± 0.2101 | 1.079 ± 0.2606 | 1.012 ± 0.2906 |
| LDL-C, mmol/L | 2.743 ± 0.5027 | 2.506 ± 0.7083 | 2.357 ±0.7886 |
| APOA1, g/L | 1.230 ± 0.1827 | 1.195 ± 0.2318 | 1.168 ± 0.2277 |
| APOB, g/L | 1.022 ± 0.1665 | 0.9596 ± 0.2289 | 0.9238 ± 0.2672 |
| Lp(a), mg/L | 201.6 ± 214.7 | 167.1 ± 136.5 | 200.5 ± 211.0 |
|  |  |  |  |
| All human subjects were recruited in the Xiamen Cardiovascular Hospital of Xiamen University in July, 2024 and February, 2025. All human subjects were Han Chinese. Data are expressed as Mean ± SD.  Abbrevations: FBG, fasting blood glucose; LA, lactate in plasma; AIP, atherosclerotic index of plasma; TC, total cholesterol; TG, triglyceride; HDL-C, high-density lipoprotein cholesterol; LDL-C, low-density lipoprotein cholesterol; APOA1, apolipoprotein A1; APOB, apolipoprotein B; Lp(a), lipoprotein a. | | | |
|  | | | |

| Supplementary Table 3. Antibodies and drugs list | | | | | | | |
| --- | --- | --- | --- | --- | --- | --- | --- |
| Target | | **Company** | **Catalog** | | **Application** | | **Dilution ratio** |
| GAPDH | | ABclonal | AC033 | | WB | | 1:10000 |
| β-Actin | | ABclonal | AC004 | | WB | | 1:5000 |
| Histone H3 | | ABclonal | A2348 | | WB | | 1:3000 |
| p21 | | ABclonal | A1483 | | WB | | 1:1000 |
|  |  | Servicebio | GB15153 | | IF | | 1:200 |
| OLR1 | | ABclonal | A1639 | | WB | | 1:1000 |
| CD36 | | ABclonal | A21716 | | WB | | 1:500 |
| Src | | ABclonal | A0324 | | WB | | 1:1000 |
| STAT3 | | Proteintech | 10253-2-AP | | ChIP | | 1:100 |
| phospho-Src-Y419 | | ABclonal | AP1027 | | WB | | 1:1000 |
| ERK1/2 | | ABclonal | A16686 | | WB | | 1:1000 |
| phospho-ERK1/2 | | ABclonal | AP0974 | | WB | | 1:1000 |
| AKT1 | | ABclonal | A17909 | | WB | | 1:1000 |
| phospho-S473-AKT1 | | ABclonal | AP0637 | | WB | | 1:500 |
| AMPKα1 | | ABclonal | A1229 | | WB | | 1:1000 |
| phospho-AMPKα1-T183 | | ABclonal | AP1441 | | WB | | 1:1000 |
| HCAR1 | | ABclonal | A20321 | | WB | | 1:1000 |
| p16 | | Abcam | ab241543 | | WB | | 1:1000 |
| GPR132 | | Santa Cruz | sc-137112 | | WB | | 1:500 |
|  |  | Proteintech | 17026-1-AP | | IHC | | 1:100 |
| F4/80 | | Servicebio | GB113373 | | IF | | 1:200 |
| MCT1 | | ABclonal | A27270 | | WB | | 1:1000 |
| MCT2 | | ABclonal | A12386 | | WB | | 1:1000 |
| MCT4 | | ABclonal | A10548 | | WB | | 1:1000 |
| WB: Western blotting; IHC: immunohistochemistry; IF: immunofluorescence. | | | | | | | |
|  | | | | | | | |
|  | **Drug** | | | **Company** | | **Catalog** | |
|  | 2-DG | | | MCE | | HY-13966 | |
|  | Oxamate | | | MCE | | HY-W013032A | |
|  | AZD3965 | | | MCE | | HY-12750 | |
|  | Sulfo-N-succinimidyl oleate sodium | | | MCE | | HY-112847A | |
|  | Quercetin | | | MCE | | HY-18085 | |
|  | Paclitaxel | | | MCE | | HY-B0015 | |
|  | Saracatinib | | | MCE | | HY-10234 | |
|  | ONC212 | | | MCE | | HY-111343 | |
|  | Streptozocin | | | Yeasen | | 60256ES80 | |
|  | Citrate | | | Sangon Biotech | | A100529 | |
|  | Lactate | | | Sangon Biotech | | A504045 | |
|  | Aldometanib | | | TargetMol | | T60122 | |
|  | 3, 5-DHBA | | | APExBio | | B7686 | |
|  | 7ACC1 | | | MCE | | HY-D0067 | |
|  | AR-C155858 | | | Aladdin | | A125208 | |
|  | Phorbol 12-myristate 13-acetate | | | MCE | | HY-18739 | |


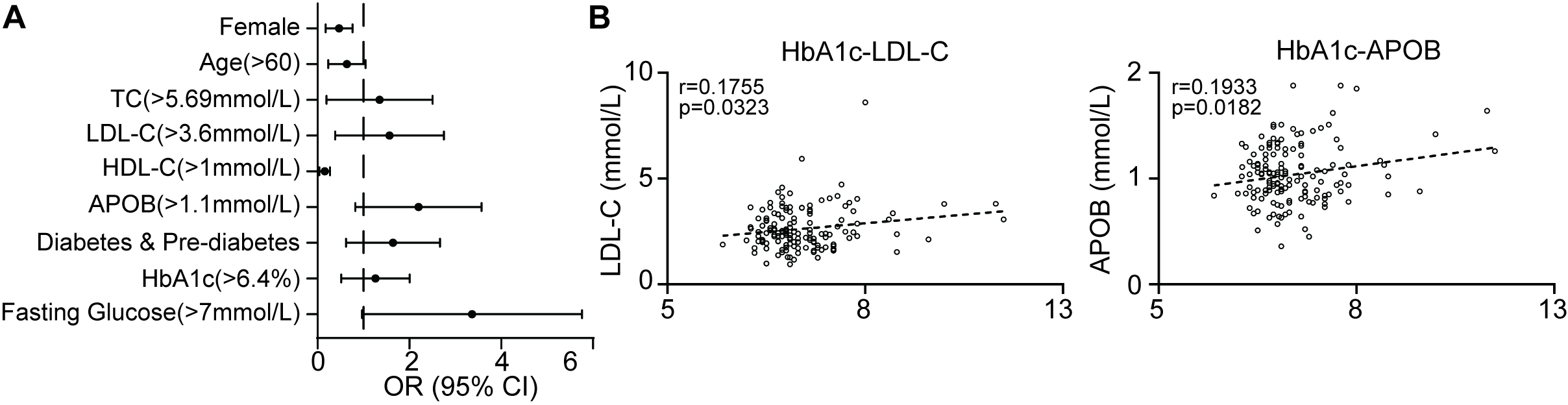


**Supplementary Figure 1. Supplemental clinical data analysis of Figure 1.**

(**A**) Forest plots depict the association of a series of index with coronary arteriosclerosis. Data are means and 95% CIs. The means were compared by independent samples t tests and proportions by χ2 tests.

(**B**) Correlation analysis of HbA1c and the levels of LDL-C and APOB. The correlations were analyzed with linear regression.


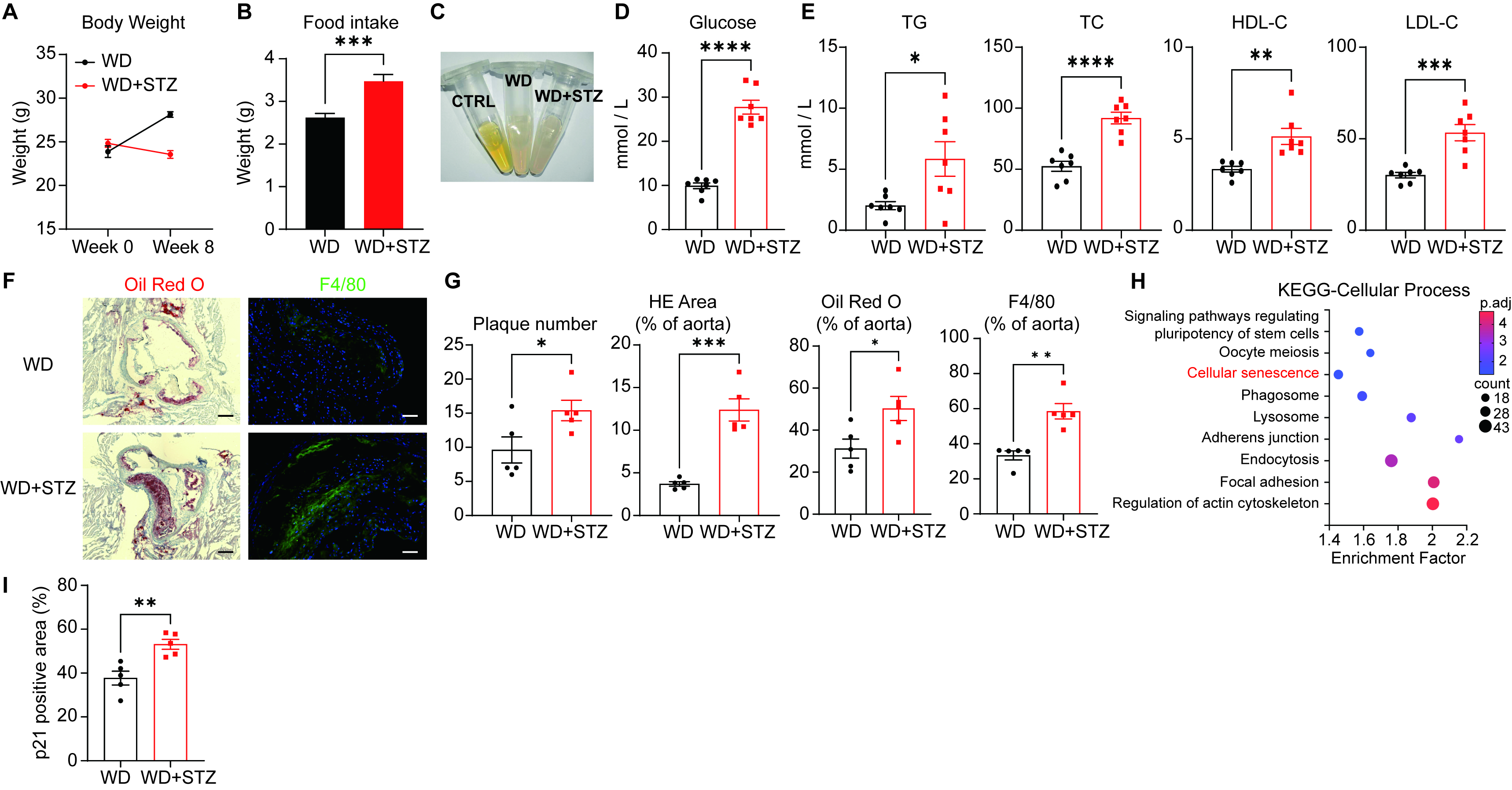


**Supplementary Figure 2. Index of the diabetic APOE^-/-^ mice.**

**(A)** Body weight of the mice at the beginning and end point of the experiment.

**(B)** Average food intake of the mice.

**(C)** Representative images of the plasma of the mice in **Figure 1D**.

**(D)** The levels of glucose in the serum of the mice. n=7 for each group.

**(E)** The levels of TC, LDL-C, HDL-C, TG in the serum of the mice. n=8 for each group.

**(F)** Representative images of the arterial roots with Oil Red O staining and F4/80 staining.

**(G)** Quantification of the plaque numbers, plaque area (H&E stained and Oil Red O stained) and the macrophage area (F4/80 positive) of **Figure 1D&S1F**. n=5 for each group.

**(H)** Bubble charts to show the cellular process changes by KEGG analysis of the differently expressed genes.

**(I)** The quantification of the p21^+^ area of **Figure 2E**. n=5 for each group.

All the quantitative data were analyzed with student t-test. *P < 0.05; **P < 0.01;***P < 0.001.


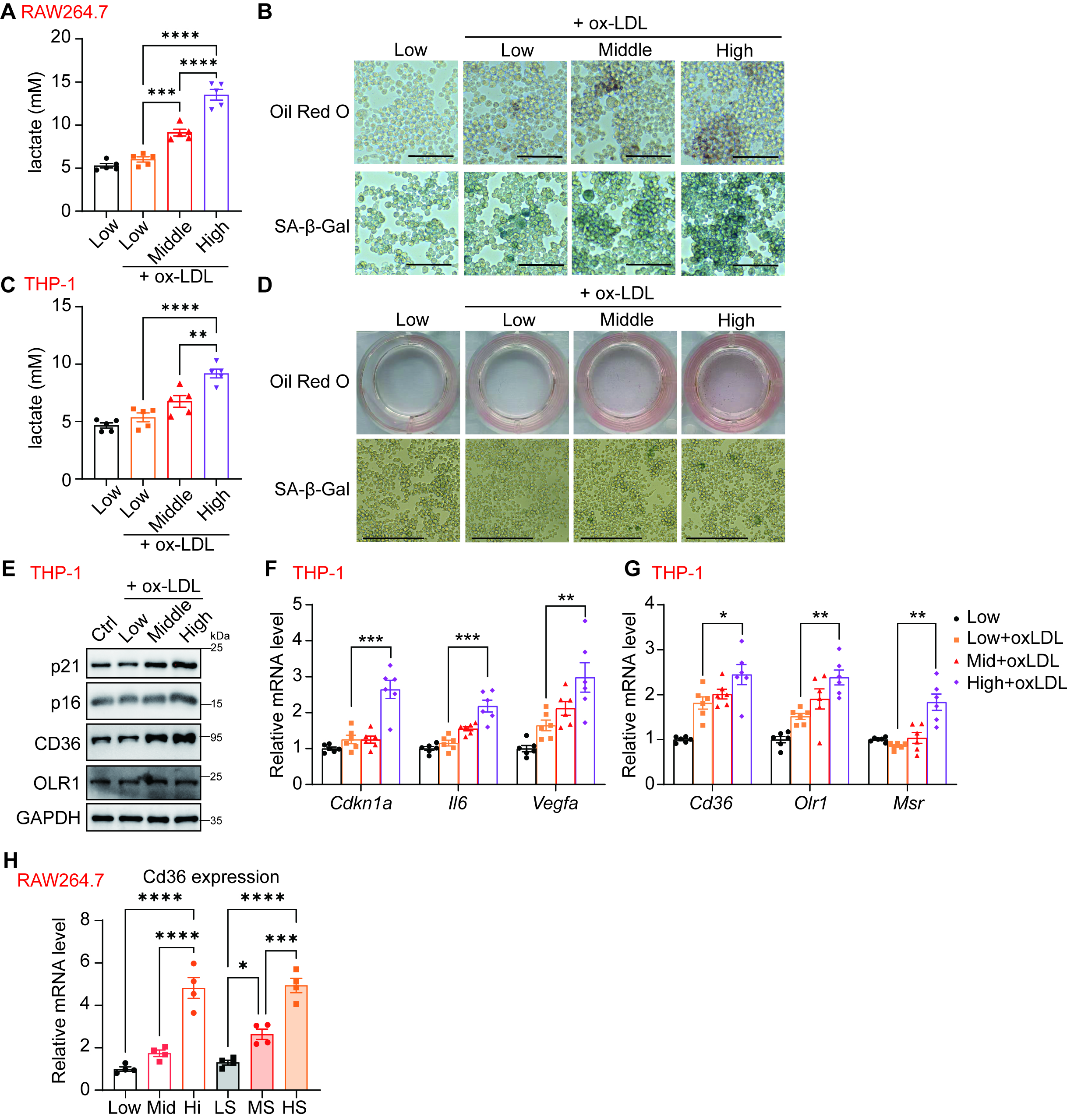


**Supplementary Figure 3. High glucose level promotes senescence in THP-1 cells.**

**(A)** Lactate concentration in the medium of the RAW264.7 cells in **Figure 2G**. n=4 for each group.

**(B)** Representative images of Oil Red O and β-Gal stained RAW264.7 cells in **Figure 2G** under light microscopy.

**(C)** Lactate concentration in the medium of the THP-1 cells. Cells were pre-treated with DMEM with different concentration of glucose for 24 hours and then treated with 40mg/mL ox-LDL for 24 hours. n=4 for each group.

**(D)** Representative images of Oil Red O and β-Gal stained THP-1 cells in **C**.

**(E)** Western blotting to detect p21, p16, OLR1 and CD36 in the THP-1 cells as in **C**.

(**F-G**) Relative mRNA levels of lipid uptake genes (**E**) and SASP genes (**D**) in the THP-1 cells as in **B**. n=6 for each group.

(**H**) Relative mRNA levels of Cd36 in the RAW264.7 cells as in **Figure 3A**. n=4 for each group.

The quantitative data were analyzed with one-way ANOVA. *P < 0.05; **P < 0.01;***P < 0.001, ****P < 0.0001.


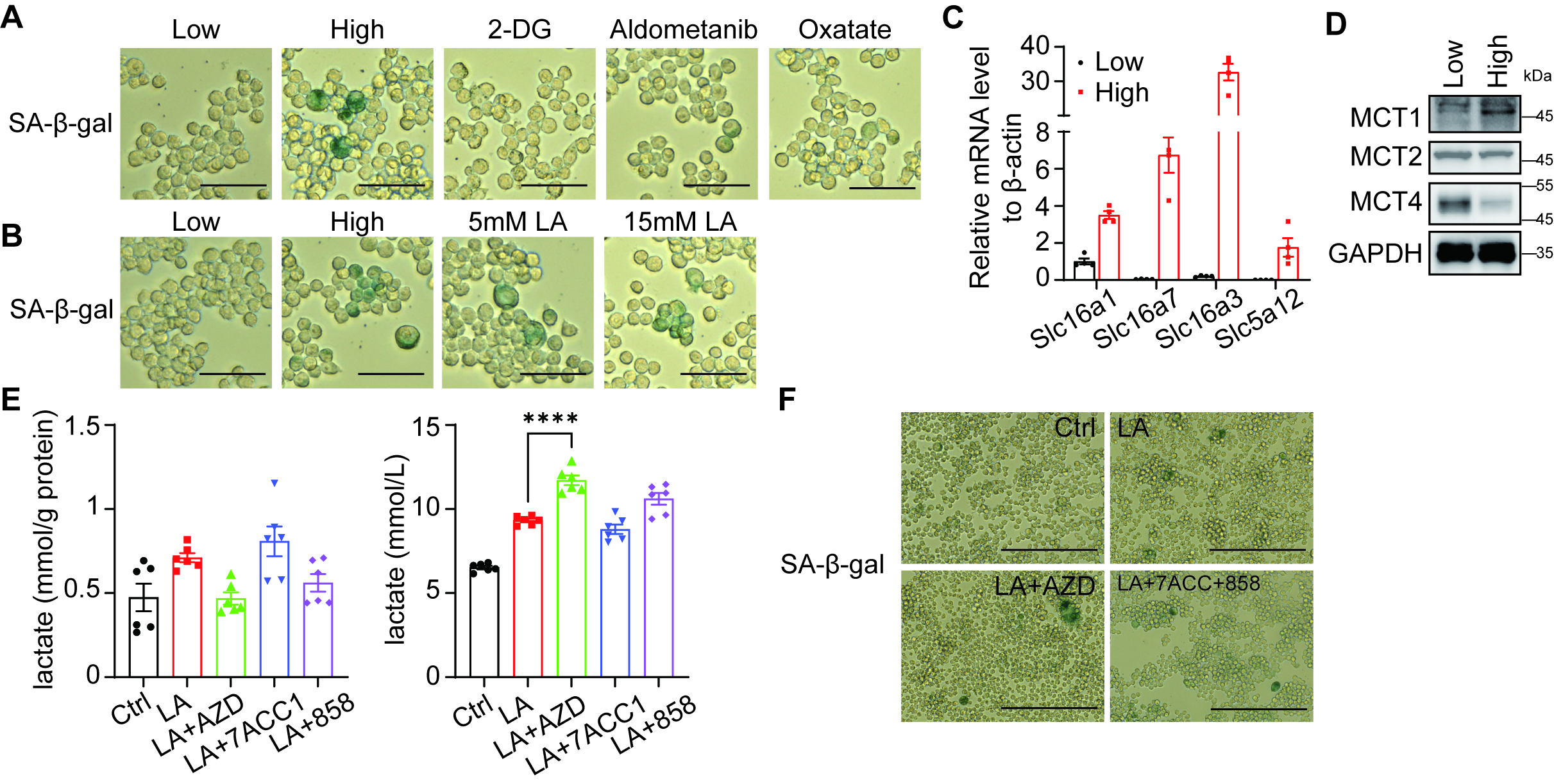


**Supplementary Figure 4. Lactate transports in macrophages.**

**(A)** Representative images of β-Galactosidase stained RAW264.7 cells in **Figure 4C** under light microscopy. Scale bar = 100μm.

**(B)** Representative images of β-Galactosidase stained RAW264.7 cells in **Figure 4E** under light microscopy. Scale bar = 100μm.

**(C)** The mRNA levels of Slc16a1 (MCT1), Slc16a7 (MCT2), Slc16a3 (MCT4), and Slc5a12 (SMCT2) in RAW264.7 cells under different levels of glucose. n=4 for each group.

**(D)** Western blotting to detect MCT1, MCT2 and MCT4 in RAW264.7 cells under different levels of glucose.

**(E)** The lactate levels inside (left panel) and outside (right panel) RAW264.7 cells with lactate and different inhibitors treatment for 24 hours. n=6 for each group. The quantitative data were analyzed with one-way ANOVA. ****P < 0.0001.

**(F)** Representative images of β-Galactosidase stained RAW264.7 cells in **Figure 4J** under light microscopy. Scale bar = 200μm.


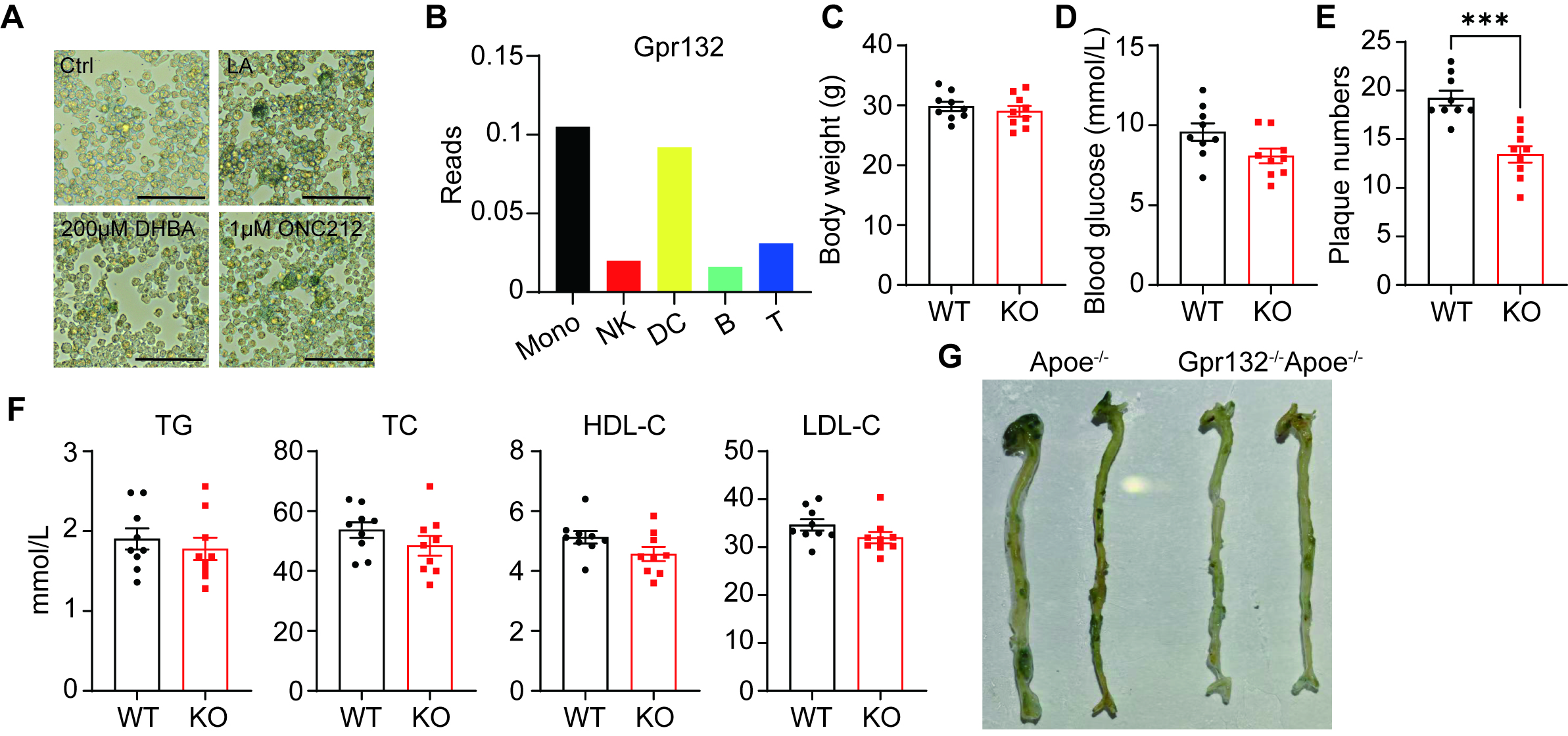


**Supplementary Figure 5. The indexes of Gpr132^-/-^ mice.**

**(A)** Representative images of β-Galactosidase stained RAW264.7 cells in **Figure 5F** under light microscopy. Scale bar = 200μm.

**(B)** The expression of Gpr132 in different cells of peripheral blood from healthy people according to GSE165816. Mono, monocytes/macrophages; NK, natural killer cells; DC, dendritic cells; B, B cells; T, T cells.

**(C)** Body weight of the mice at the end of experiments as in **Figure 5**. n=9 for each group. WT, Apoe^-/-^ mice; KO, Gpr132^-/-^Apoe^-/-^ mice.

**(D)** The levels of fasting blood glucose of the mice. n=9 for each group.

**(E)** The quantification of plaque numbers of the aorta in **Figure 5I**. The quantitative data were analyzed with student t-test. ***P < 0.001.

**(F)**The levels of serum lipid index of the mice. n=9 for each group.

(**G**) Representative images of β-Gal stained aorta.


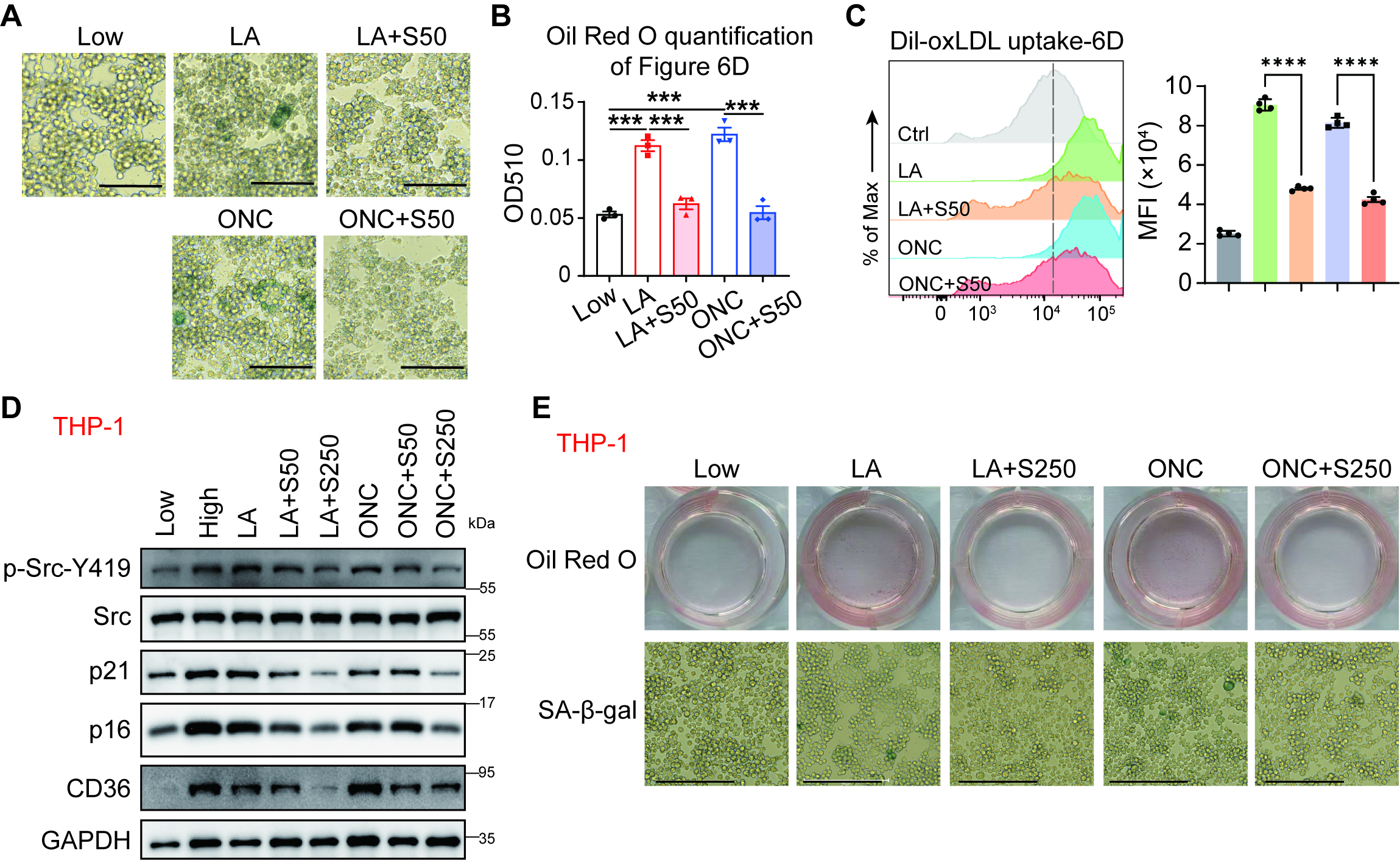


**Supplementary Figure 6. The effects of Saracatinib in RAW264.7 and THP-1 cells.**

**(A)** Representative images of β-Galactosidase stained RAW264.7 cells in **Figure 6C** under light microscopy. Scale bar = 100μm.

**(B)** Quantification of Oil Red O in **Figure 6D**. n=3 for each group.

(**C**) Flow cytometry analysis of Dil-ox-LDL uptake by the RAW264.7 cells as in **Figure 6C&D.**

**(D)** Western blotting to detect the phosphorylation of Src, p16, p21, and CD36 in the THP-1 cells with DMEM with 1.5mg/mL glucose (Low), 4.5mg/mL glucose (High), 1.5mg/mL glucose with 10mmol/L lactate (LA), 1.5mg/mL glucose with 1μmol/L of ONC212 (ONC), lactate or ONC212 with different concentration of saracatinib for 24 hours.

**(E)** Representative images of β-Gal and Oil Red O stained THP-1 cells in **D**.


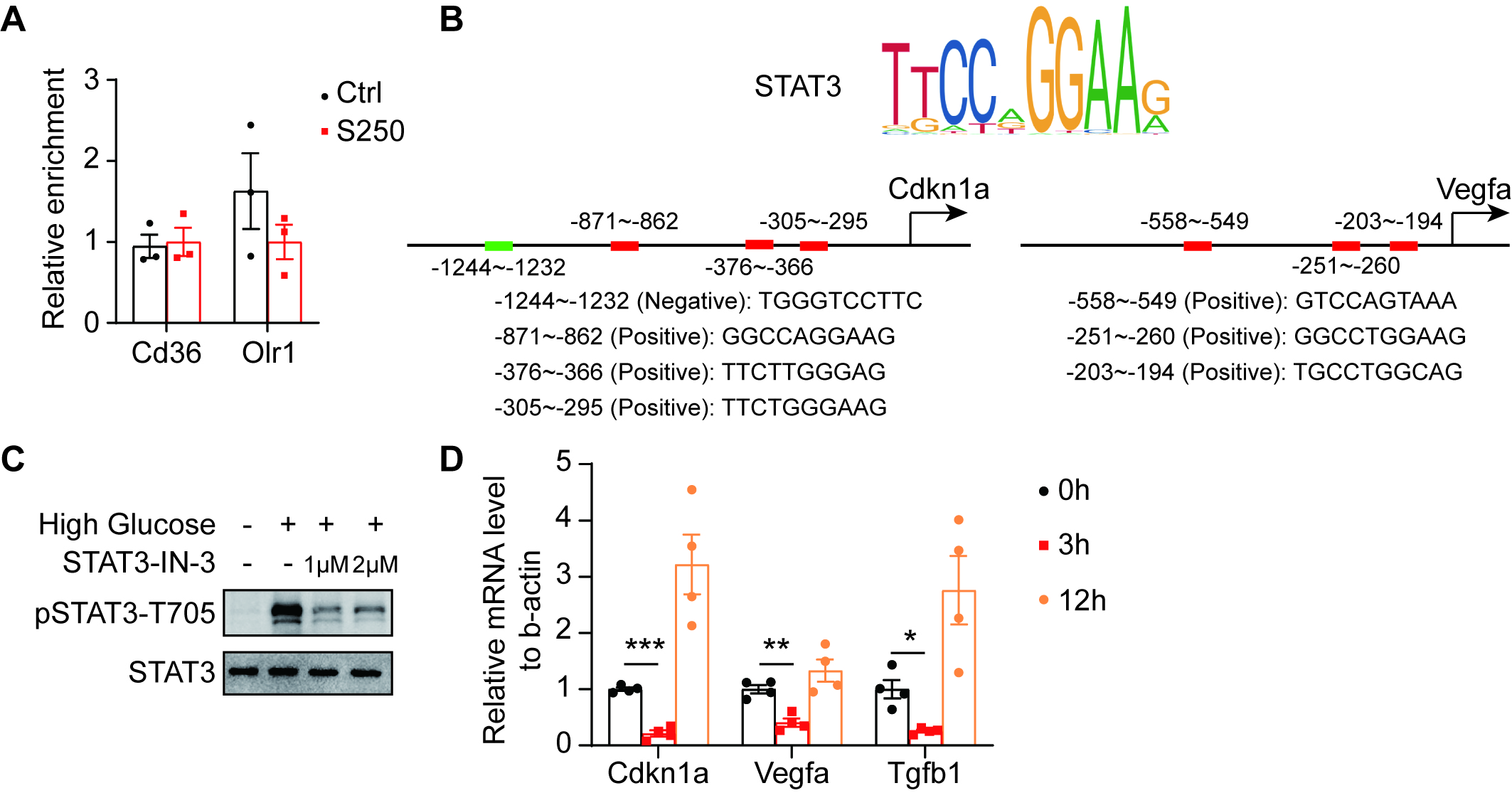


**Supplementary Figure 7. The transcriptional activity of STAT3.**

**(A)** ChIP-qPCR performed with anti-STAT3 antibody in RAW264.7 treated by 4.5mg/mL glucose with DMSO (Ctrl) or 250nM saracatinib (S250) for 24 hours and then stimulation with 40mg/mL ox-LDL for 12 hours. n=3 for each group.

**(B)** A diagram to show the binding sites of mouse STAT3 on the promoters of Cdkn1a and Vegfa, according to JASPAR.

(**C**) Western blotting to detect the phosphorylation of STAT3 in the RAW264.7 cells with the treatment of STAT3-IN-3 for 1 hour after 24-hour stimulation with 40mg/mL oxLDL.

**(D)** Relative mRNA levels of Cdkn1a, Vegfa, and Tgfb1 in the RAW264.7 cells pre-treated with 4.5g/L glucose and 40mg/mL oxLDL for 24 hours, followed by 1μM STAT3-IN-3 treatment for different time. n=4 for each group. The quantitative data were analyzed with one-way ANOVA. *P < 0.05; **P < 0.01;***P < 0.001, ****P < 0.0001.


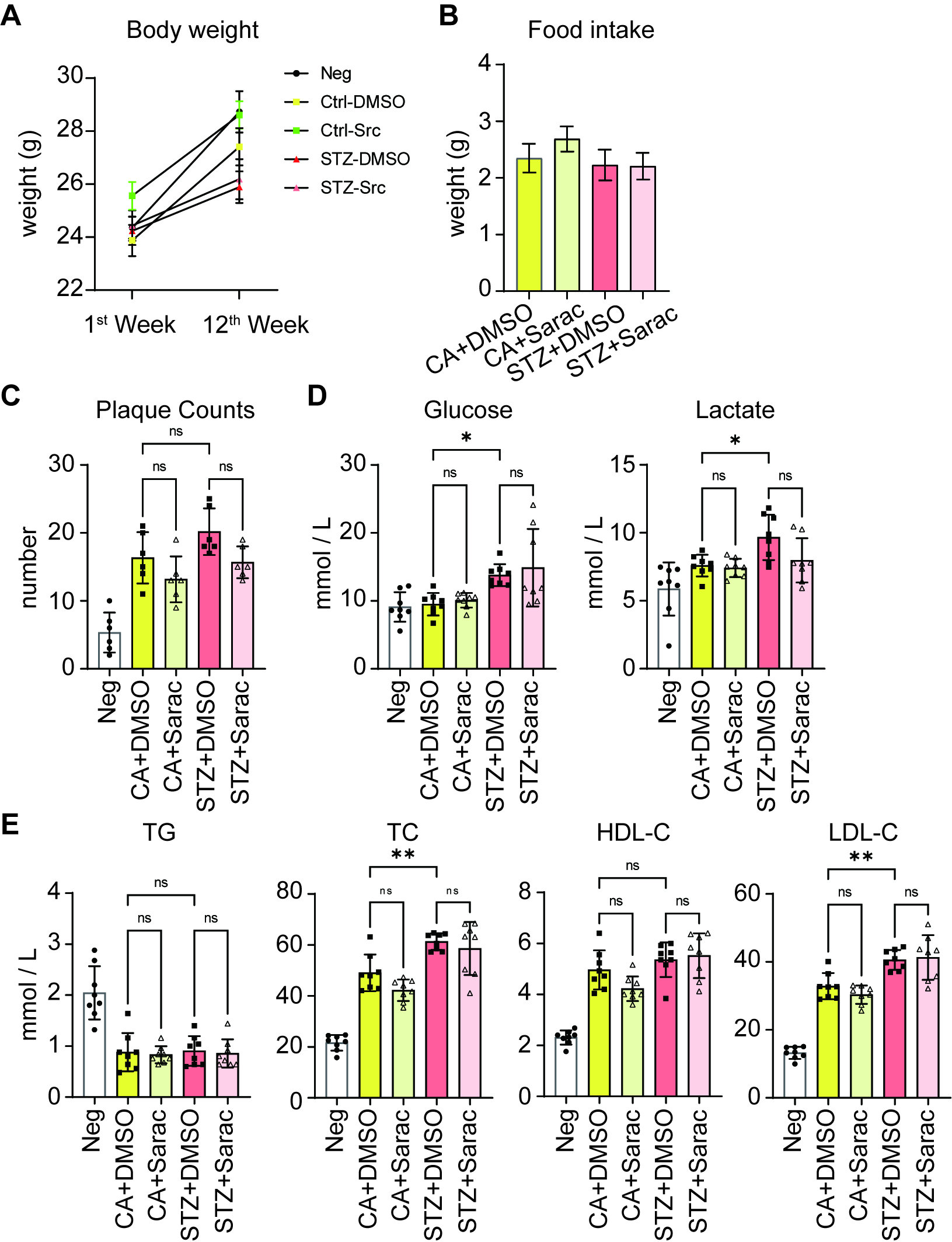


**Supplementary Figure 8. Index of the diabetic APOE^-/-^ mice as in Figure 6.**

**(A)** Body weight of the mice at the beginning and end point of the experiment.

**(B)** Average food intake of the mice.

**(C)** The plaque number of Oil Red O stained aorta as in **Figure 6G**. n=6 for each group.

**(D-E)** The levels of fasting blood glucose, serum lactate (**D**) and serum lipid indexes (**E**) of the mice as in **Figure 6**. n=8 for each group.

All the quantitative data were analyzed with one-way ANOVA. *P < 0.05; **P < 0.01.


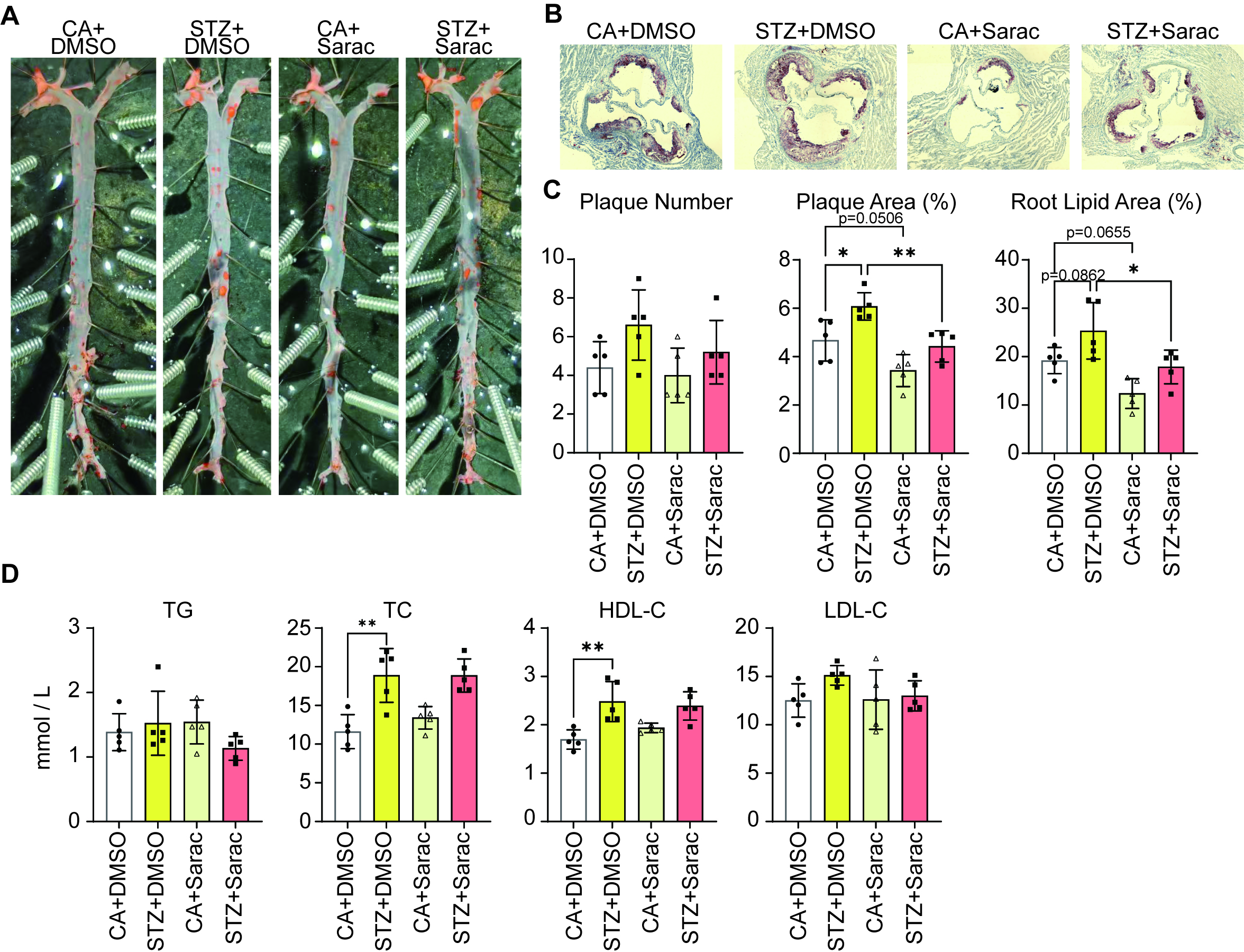


**Supplementary Figure 9. Index of the female APOE^-/-^ mice treated with saracatinib.**

(**A**) Representative images of Oil Red O stained aorta.

(**B**) Representative images of the arterial roots with Oil Red O staining.

(**C**) Quantification of the plaque number, plaque area and Oil Red O positive area of the roots. n=5 for each group.

**(D)** The levels of serum lipid indexes of the mice. n=5 for each group.

All the quantitative data were analyzed with one-way ANOVA. *P < 0.05; **P < 0.01.


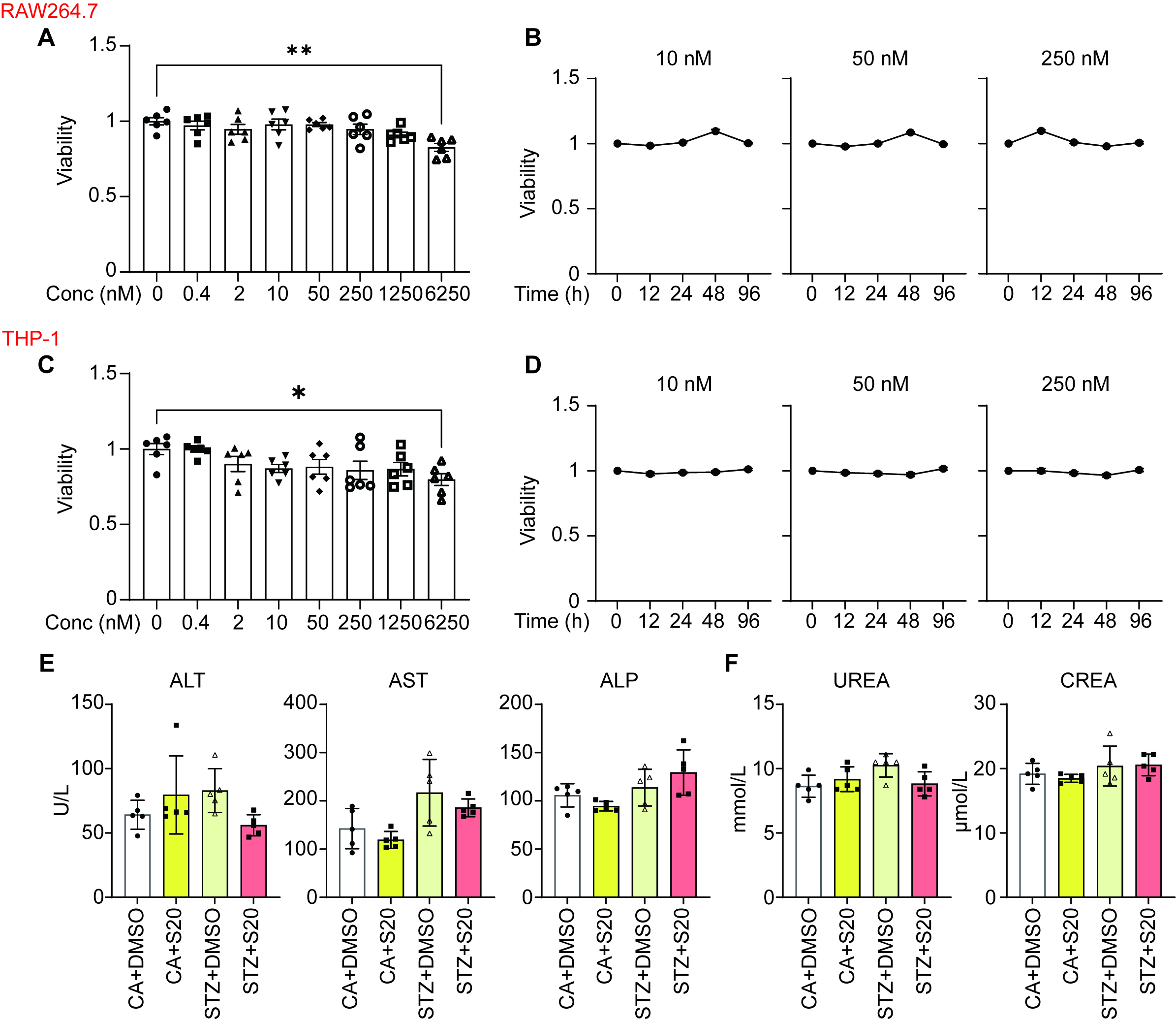


**Supplementary Figure 10. The toxicity of saracatinib.**

(**A**) Viability of RAW264.7 cells treated with different dose of saracatinib for 24 hours. n=6 for each group.

(**B**) Viability of RAW264.7 cells treated with 10, 50, 250nM saracatinib for different time. n=6 for each group.

(**C**) Viability of THP-1 cells treated with different dose of saracatinib for 24 hours. n=6 for each group.

(**D**) Viability of THP-1 cells treated with 10, 50, 250nM saracatinib for different time. n=6 for each group.

**(E)** The levels of serum ALT, AST and ALP of the mice as in **Figure 6**. n=5 for each group.

**(F)** The levels of serum Urea and creatinine of the mice as in **Figure 6**. n=5 for each group.

All the quantitative data were analyzed with one-way ANOVA. *P < 0.05; **P < 0.01.
